# Developmental deglutition and intrinsic tongue muscle maturation phenotypes in the Ts65Dn mouse model of Down syndrome

**DOI:** 10.3389/fneur.2024.1461682

**Published:** 2024-12-11

**Authors:** Tiffany J. Glass, Benjamin A. Chatwin, Erin H. Fisher, Kabao K. Hang, Qiuyu Yang, Riley Brutto, Rohan Waghray, Nadine P. Connor

**Affiliations:** ^1^Department of Surgery, Division of Otolaryngology, University of Wisconsin, Madison, WI, United States; ^2^Department of Surgery, Statistical Analysis and Research Programming Core, University of Wisconsin, Madison, WI, United States; ^3^Department of Communication Sciences and Disorders, University of Wisconsin, Madison, WI, United States

**Keywords:** Down syndrome, Ts65Dn, mouse, intrinsic tongue, feeding, deglutition, maturation, weaning

## Abstract

**Introduction:**

Down syndrome (DS) is associated with difficulties with feeding during infancy and childhood. Weaning, or transitioning from nursing to independent deglutition, requires developmental progression in tongue function. However, little is known about whether postnatal tongue muscle maturation is impacted in DS. This study tested the hypothesis that the Ts65Dn mouse model of DS has developmental delays in deglutition, comprised of differences in eating and drinking behaviors relative to euploid controls, coinciding with atypical measures of intrinsic tongue muscle microanatomy.

**Methods:**

The Ts65Dn mouse model of DS and euploid controls were evaluated at 7 days of age (p7; nursing), p21 (weaning), and p35 (mature deglutition) (n = 13–18 mice per group). Eating behavior, drinking behavior, and body weight changes were quantified in p21 and p35 mice through the use of automated monitoring over 24 h. Intrinsic tongues of mice at all three ages were sectioned and stained to permit quantification of the sizes of the four major intrinsic tongue muscles. Transverse intrinsic tongue muscles were evaluated for myofiber size (average myofiber cross sectional area (CSA) of all fibers, MyHC2a fibers, MyHC 2b fibers, and minimum Feret fiber diameter), and percentage of MyHC isoforms (%MyHC2a + fibers, and %MyHC 2b + fibers) in anterior, middle, and posterior regions.

**Results:**

Ts65Dn showed significant differences from euploid in deglutition measures. Compared to euploid, Ts65Dn also showed differences in intrinsic tongue muscle microanatomy and biology. Specifically, Ts65Dn intrinsic tongues had smaller transverse muscle myofiber size measures than control in the anterior and middle tongue, but not in the posterior tongue.

**Conclusion:**

Differences in intrinsic tongue muscles coincide with feeding phenotypes in the Ts65Dn mouse model of DS.

## 1 Introduction

Down syndrome (DS) is a developmental disorder in which atypical tongue movement or positioning can contribute to impairments of speech (1–3), breathing (4), feeding (5, 6), and swallowing (7). These challenges can alter health, communicative function, and quality of life (8, 9). There are currently relatively few evidence-based treatments for developmental deficits associated with tongue function in DS, and despite a longstanding awareness that differences in tongue movement can occur in DS, postnatal maturation of the lingual system in DS has rarely been studied from a basic biological standpoint.

The tongue is a complex muscle system comprised of several different interdigitating muscles, and early childhood is a time of rapid change and growth in both oromotor functions involving the tongue (10), as well as in the anatomy that is integral to these functions (11). The four primary muscles comprising the intrinsic tongue are the superior longitudinal (SL), inferior longitudinal (IL), transverse, and verticalis (12). The SL acts to raise the tongue tip and shape the superior tongue surface into a concavity. The IL acts to lower the tongue tip and shape the superior tongue surface into a convexity. The IL and SL can also shorten the tongue (12). The verticalis flattens the tongue, while the transverse muscle narrows and elongates the tongue. In humans, these muscles work in concert to help carry out the very complex and rapid lingual movements involved in speech and feeding (13, 14). However, it is unclear how each of these four intrinsic tongue muscles mature after birth in a way that coincides with the postnatal acquisition of the discrete tongue muscle capabilities at the ages in which expansion of tongue functions for speech development and feeding are achieved. While DS is strongly associated with impairments of speech and feeding, and differences of intrinsic tongue and muscle biology have sometimes been reported to occur in DS (15, 16), it is unclear whether and how maturation of intrinsic tongue muscles are atypical in DS.

Skeletal muscles encompass considerable biological diversity, including a wide range of muscle-specific functional properties (17). In typical post-natal development, these properties change over time with activity levels and age (18). Myosin heavy chain (MyHC) proteins are functionally integral to muscle contraction. MyHC isoforms are indicators of muscle fiber type, with MyHC 2a, 2x, and 2b prevalent in fast-contracting myofibers, and MyHC I present in slow myofibers. Of these MyHC isoforms, MyHC 2b may be expressed in murine tongue myofibers that are fastest-contracting, but may also have less endurance, or greater fatiguability. A diversity of MyHC expression profiles occurs in muscles throughout the body and distinct MyHC profiles in muscles are sometimes used as indirect indicators of muscle fiber types, contractile properties, and muscle fatiguability (19, 20).

In typical development, MyHC profiles of cranial muscles correlate with different stages of postnatal oromotor maturation (21–25). The tongue undergoes significant alterations of the MyHC profiles before and after weaning (26–28). Analysis of gross anatomical subdivisions of the intrinsic tongue in young mice has suggested that not only does the biology of the intrinsic tongue change with weaning, but that different tongue regions change in different ways during this time period (26, 27). Further, a prior study in humans with DS is compatible with the possibility that the tongue in DS may contain altered proportions of fast myofibers relative to typical controls (15, 29). However, it remains to be confirmed in controlled studies that DS causes significant differences in tongue MyHC profiles. Furthermore, the maturational course of MyHC phenotypes in the tongue in DS is unclear.

Mice have several fundamental attributes of tongue form and function similar to those of humans (30, 31). Mice are also amenable to translational functional outcome measures that have correlates to clinical measures used to quantify dysfunction of tongue movement in humans (32, 33). Experience in prior studies indicates 1 week, 3 weeks, and 5 weeks of age in mice correspond to suckling, weaning, and mature oromotor function (21, 34). Efforts to standardize murine research for pre-clinical studies of muscle function have identified these ages in mice to correspond to human developmental ages of early infancy, 6 months, and 10–11 years, respectively (35, 36). Quantification of functional and biological measures of the intrinsic tongue at these timepoints may provide a standardized translational framework for evaluation of typical and atypical lingual maturation. Ideally, this could support improved mechanistic explanations of normal lingual developmental milestones and greater experimental precision of studies investigating the muscular underpinnings of disorders in which maturation of tongue movement is delayed, aberrant, or absent.

The Ts65Dn mouse model of DS has been a widely-used model that recapitulates several phenotypes of DS, including craniofacial, motor, and swallowing phenotypes (37–41). Ts65Dn has previously been shown to demonstrate a variety of phenotypes applicable to feeding and swallowing differences associated with DS in humans (39, 42). Therefore, the current study used the Ts65Dn mouse model of DS and sibling euploid controls to test a central hypothesis that DS is associated with developmental delays in maturation of the tongue neuromuscular system. Specifically, it was hypothesized that DS would cause delays in acquisition of eating and drinking skills at p21 and p35, coinciding with genotype-specific alterations of MyHC profiles in the intrinsic tongue. Both functional oromotor and tongue tissue analysis measures were used to evaluate tongue maturation at discrete postnatal developmental timepoints representative of nursing, weaning, and mature deglutition, to define aspects of tongue function characteristic of typical oromotor development in mice, as well as to reveal how DS affects maturation of these muscles. Automated home-cage monitoring technology was used to collect multiple measures of eating and drinking behavior of mice continuously for 24 h to provide indicators of tongue muscle function, and comprehensive microscopy of intrinsic tongue muscles at the ages studied permitted evaluation of microanatomical measures indicative of tongue muscle biology. In this way, both functional and biological measures of the intrinsic tongue were evaluated during a time period of rapid oromotor development in a mouse model of a developmental disorder associated with pediatric feeding challenges.

## 2 Methods

### 2.1 Mice

**Figure 1:**
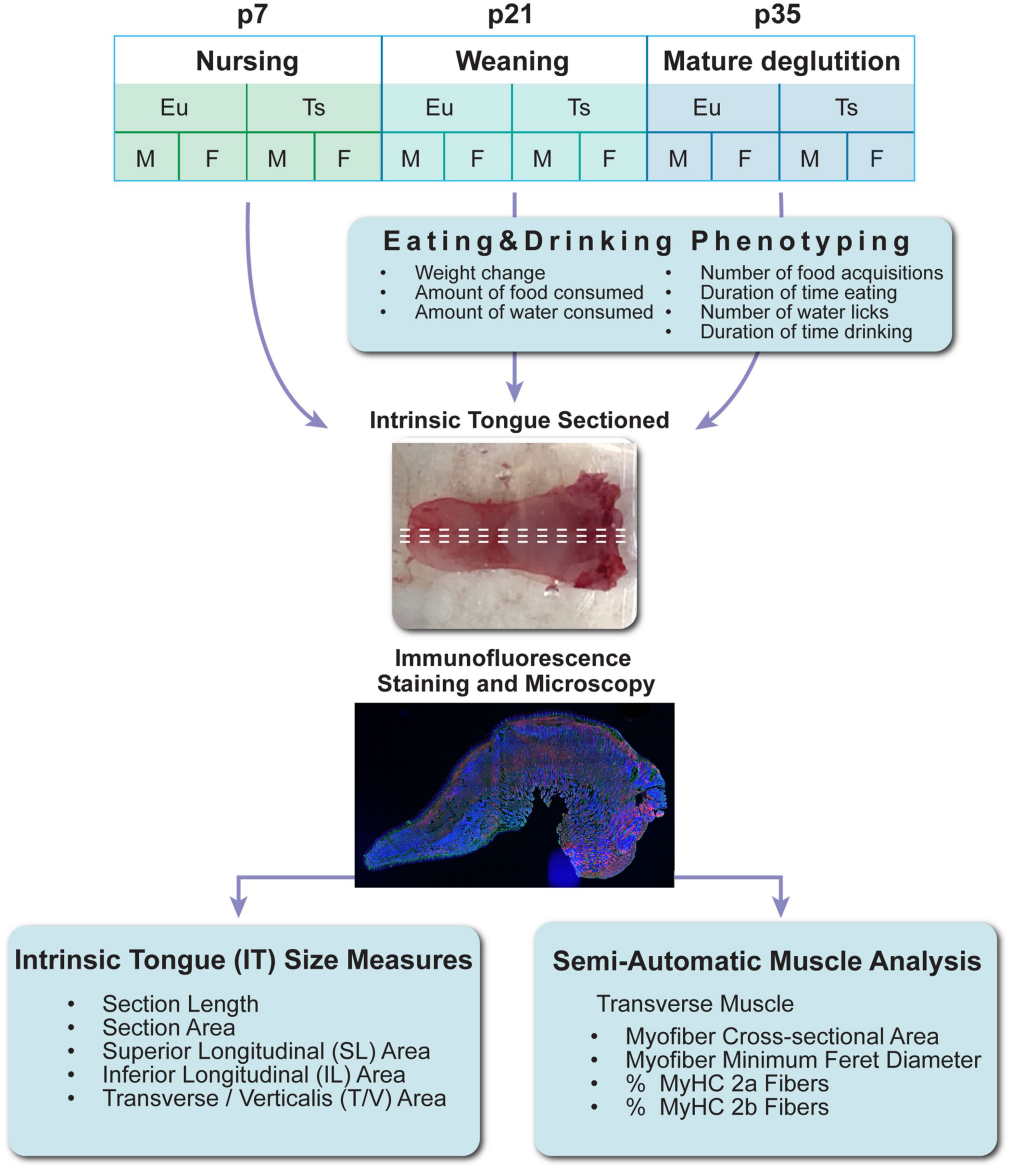
Experiment design. Eu = euploid, Ts = Ts65Dn, p = postnatal day of age, M = male, F = female, MyHC = Myosin Heavy Chain Isoform.

All live animal work was performed in accordance with a protocol approved by the University of Wisconsin School of Medicine and Public Health institutional animal care and use committee (IACUC). Experimental mice were generated from breeding pairs of Ts65Dn mice obtained from Jackson Laboratories, comprised of either a female Ts65Dn mouse (stock number 005252) mated to a male euploid control C57BL/6JEiJxC3Sn.BLiA-Pde6b+/(DnJ)F1 mouse (stock number 003647), or a female Ts65Dn mouse (005252) mated to a euploid male control mouse from the same stock number (005252). Mice were genotyped through Transnetyx. To reduce risks for investigator bias, mice were assigned alphanumeric identifier codes prior to genotyping, and alphanumeric codes were used throughout the study to identify mice, tissue samples, and data files. Mice were housed in standard microisolator cages with dimensions of 7 × 5 × 11 inches, at a density ranging from 2 to 4 weaned mice per cage, and were kept on a reverse light cycle with the dark period from 9:00 am to 9:00 pm. Room temperature and humidity were facility regulated to maintain a range of 68–79°F and 30–70%, respectively. All breeding mice and progeny throughout the study were maintained on Harlan Teklad chow diet #7913 and had *ad libitum* access to water bottles with metal spouts. Breeding pairs were monitored daily for new litters. Litter sizes averaged 5 pups per litter but ranged from 1 to 11 pups. No mice were excluded from the study due to litter size. As described in Figure 1, mice from litters were arbitrarily assigned to either euthanasia and tissue collection at postnatal day (p)7, p21-22, or p35-36, or else automated behavioral phenotype analysis at p21 or p35 followed by euthanasia and tissue collection the following day. For euthanasia and tissue collection, mice were euthanized through CO2 at p21-22, or p35-36. Mice at p7 were euthanized through CO2 followed by decapitation, or through acute hypothermia and a cardiac injection of Euthasol. Following euthanasia, mice were weighed, and tongues were removed in their entirety at the level of the hyoid bone, frozen in Optimum Cutting Temperature (OCT) compound in plastic cassettes and stored at −80°C prior to further processing. Tongue muscles were not stretched or pinned during freezing, and instead were frozen in their natural state, free of physical manipulation other than proper positioning in the cassette.

### 2.2 Eating and drinking behavior phenotyping

Mice were evaluated through automated home cage eating and drinking behavioral phenotype analysis at 21–22 days of age and 35–36 days of age. In these experiments mice were provided with the same type of water and chow (Harlan Teklad chow diet #7913) as used for the standard colony maintenance. These experiments were conducted in the same animal housing room as used for general colony housing of all mice in the study, and were conducted by workers who were also involved in daily monitoring of mice for routine husbandry purposes. Automated eating and drinking phenotyping experiments were initiated during the dark cycle, between 12 pm and 4 pm. Behavioral phenotype experiments were initiated by placing mice in a Noldus PhenoTyper home cage (Noldus PhenoTyper, Wageningen, the Netherlands), with no acclimation time provided prior to the initiation of a 24-h span of data collection. The reason separate acclimation periods were not provided prior to data collection in the Noldus PhenoTyper cage is that novelty in food and hydration encounters, as well as novelty of the environment in which weaning occurs, is intrinsic to the experience of weaning for many human children. Mouse eating and drinking behavior in the Noldus PhenoTyper cage was quantified with a lickometer, feeding monitor with an infrared beam-break, and Ethovision™ XT software, versions 16 and 17 (Supplementary Figure S1). The floor of the PhenoTyper home cage was raised to ensure all mice in the study were easily able to reach the food and water without rearing on hind limbs, and the shaft of the water nozzle was insulated to ensure only contact between the mouse and the metal tip of the nozzle would activate the lickometer. Each data acquisition session for this experiment totaled 24 h of data collection comprised of eight consecutive 3-h trials. To prevent social isolation and promote animal welfare during data collection, each data acquisition session comprised of two mice of the same age [postnatal day 21 (p21) or postnatal day 35 (p35)], same sex (male or female), and same genotype (euploid or Ts65Dn) residing in the cage together. During the twenty-four-hour period, the system recorded every time one of the mice accessed food or water. Each session was manually reviewed to detect behavioral artifacts, such as activation of the food hopper beam break with a tail rather than through eating, and to manually correct for artifact in each trial period as needed. Collected measures included the frequency of food acquisition (number of times a food hopper beam break was activated), eating duration (duration of beam break), the number of water licks, and the duration of water licks, defined as the duration of time any part of the mouse, including the tongue, touched the water spout. Mouse weight, water volume, and food weight were quantified immediately before and immediately after each 24-h experiment. Mouse weight was recorded individually for each mouse, but all other data points for measures for which mice were evaluated in pairs were comprised of the pooled values of both mice in the pair.

### 2.3 Tissue sectioning and staining

A cryostat was used to section intrinsic tongues along a mid-sagittal plane of the tongue. Five to seven 10-μm sections were obtained for each intrinsic tongue sample. Tissue staining was performed as described in an on-line protocol validated for this study (43). Slides were blocked in 1% bovine serum albumin (BSA) and 0.1% Triton-X 100 in phosphate-buffered saline (PBS). Slides were incubated at 4°C overnight with Developmental Studies Hybridoma Bank (DSHB) primary antibodies mouse IgM BF-F3, mouse IgG1 SC-71, as well as PBS (these three applied at 1:1:1), and Sigma-Aldrich antibody rabbit anti-laminin (applied at 1:200). BF-F3 and SC-71 were used for the detection of myosin heavy chain isoform (MyHC) 2b and 2a, respectively. On the following day, the slides were rinsed three times with PBS and incubated at room temperature for 1 h with secondary antibodies in 10% normal goat serum (NGS) in PBS. The secondary antibodies used were goat anti-mouse IgM Alexa Fluor 350, A31552 (applied at 1:50), goat anti-mouse IgG1 Alexa Fluor 568, A21124 (applied at 1:300), and goat anti-rabbit IgG Alexa Fluor 633, A21071 (applied at 1:250). Slides were rinsed with PBS three times and coverslipped with ProLong™ Gold (Thermo Fisher Scientific) mounting media. Each staining run included mouse soleus muscle as a biological positive control for MyHC 2a and negative control for MyHC 2b, and mouse EDL muscle as a biological positive control for MyHC 2b. Each staining iteration also included technical negative control intrinsic tongue sections comprised of omission of primary antibodies. Slides were stored at 4°C prior to imaging.

### 2.4 Microscopy acquisition

An Olympus Bx53 epifluorescence microscope with a motorized stage controlled through CellSens software (Olympus) was used to image the tissue sections. For each image acquisition session, optimal image exposure settings for each fluorescence channel were determined using the biological and technical control slides. For each mouse, one intrinsic tongue section was imaged in its entirety, using a 20x objective to generate a composite image of the entire tongue section by stitching together multiple fields of view. Tongue tissue sections selected for imaging and analysis were confirmed to extend from the tip of the anterior intrinsic tongue to the end of the posterior intrinsic tongue, and to have been sectioned in a mid-sagittal plane such that transverse intrinsic tongue myofibers in cross-section alternated with verticalis muscle fibers in longitudinal orientation. Microscopy data and protocols associated with this study are available on the SPARC Portal (RRID: SCR_017041): https://doi.org/10.26275/ol8k-rau8.

### 2.5 Image analysis for intrinsic tongue muscles

**Figure 2:**
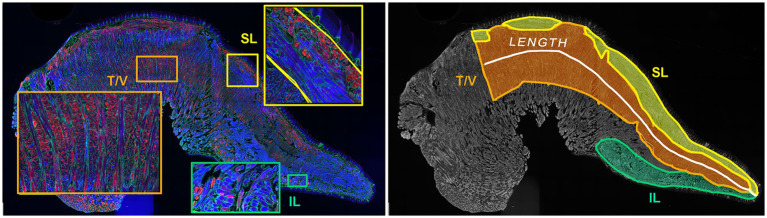
Manual image segmentation for intrinsic tongue muscle measurements. Left panel: Inset magnifications of the superior longitudinal (SL), inferior longitudinal (IL), transverse / verticalis (T/V) muscles show differences of myofiber orientation and staining properties of each muscle. Right panel: Microscopy images of intrinsic muscles were digitally segmented to permit quantification of length and muscle areas.

The program FIJI (44) was used to measure muscle section area of the superior longitudinal (SL), inferior longitudinal (IL), transverse/verticalis (T/V), and the full intrinsic tongue muscles in one tissue section from each mouse (Figure 2). Each muscle was identified within the intrinsic tongue based on its relationship to anatomical landmarks in the tongue (45) (including dorsal epithelium, ventral mucosa, lingual salivary glands, central lingual nerves and vessels) and myofiber properties such as myofiber orientation and myofiber staining characteristics. The SL muscle was identified as immediately below the dorsal tongue epithelium, and immediately superior to the T/V, and extends the length of the IT. The T/V muscle was identified as immediately inferior to the SL muscle and superior to the lingual nerve, artery, and vein. The IL muscle is immediately above the ventral tongue mucosa, and below the T/V muscle, ending posteriorly at the region the genioglossus enters the intrinsic tongue, adjacent to the frenulum. In some sections, a group of myofibers was observed above the IL and below the lingual nerve, artery, and vein. These myofibers were tentatively anticipated to be extensions of extrinsic tongue muscles or an indeterminate category and were not analyzed.

To evaluate interrater reliability of intrinsic tongue muscle size analyses, intrinsic tongue images were analyzed separately by two independent raters. Pearson correlation coefficients (r) were determined to be between 0.82 and 0.95, indicating excellent rater agreement, for the measures of intrinsic tongue length, intrinsic tongue area, T/V area, and SL area. However, the Pearson correlation coefficient of IL area was determined to be 0.62 or only moderate to fair agreement between raters. Therefore, images for which two raters substantially disagreed in identification of IL area were independently reviewed by a third experienced rater and rater discussions to confirm final values for IL area. Images in which sectioning plane deviated from midsaggital to parasaggital along the length of the section were excluded from analysis of intrinsic tongue muscle size measures.

### 2.6 Image analysis for myofibers of the transverse muscle

**Figure 3:**
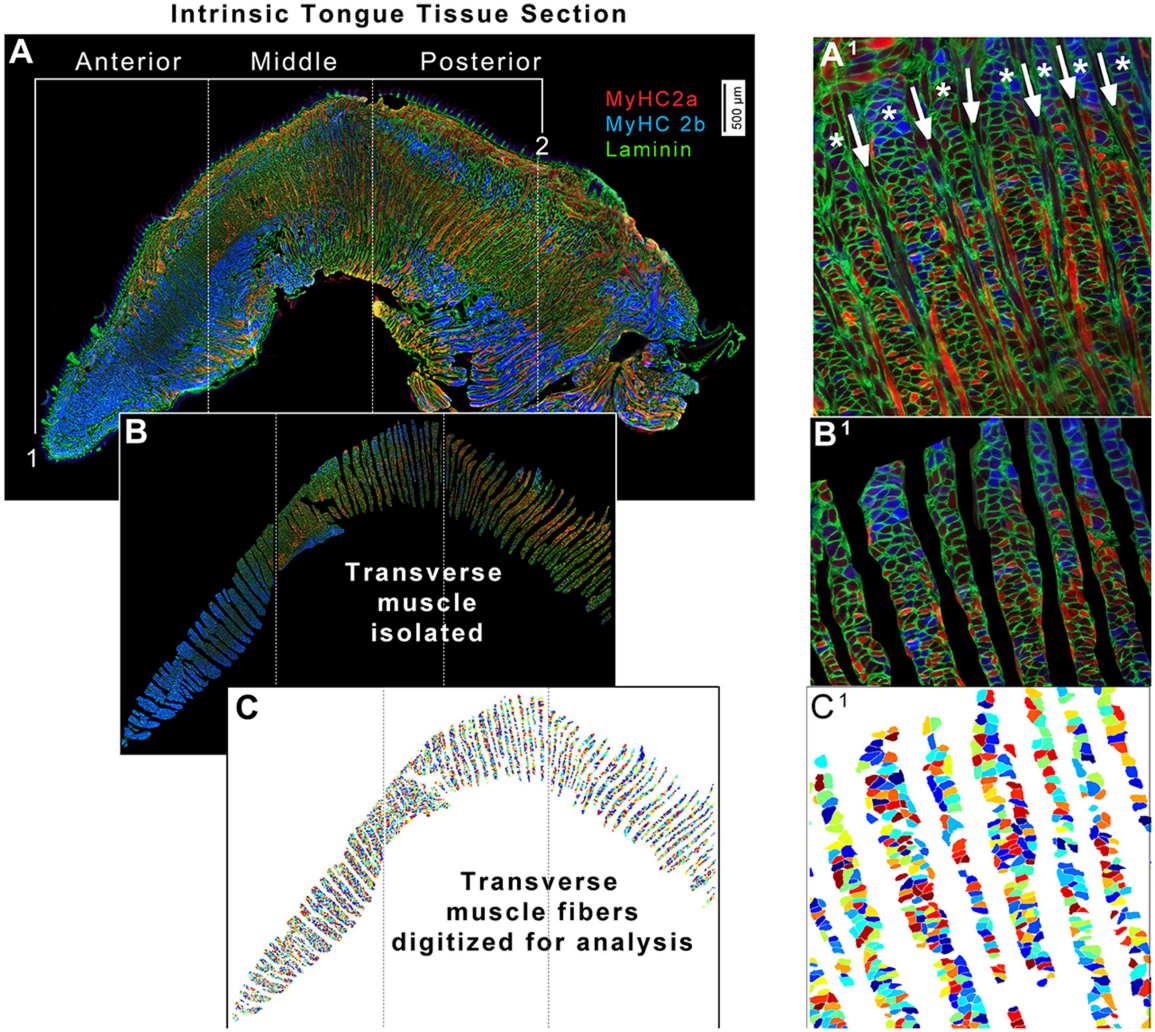
Quantitative image analysis strategy for transverse muscles from intrinsic tongue tissue sections. **(A)** Intrinsic tongue sections are imaged in their entirety. For the purposes of analyses, the anterior border of the intrinsic tongue was defined at the tongue tip (1), and the posterior border of the intrinsic tongue (2) was defined as the location where the dorsal epithelium transitioned to smooth mucosa, and/or at the location of the sublingual salivary glands. These landmarks were used to set the region which was manually divided into three image files; anterior, middle, and posterior. **(A**^**1**^**)** Zoomed image excerpt from the middle region of intrinsic tongue shown in panel **A**. Asterisks (*) indicate the transverse muscle columns, alternating with verticalis muscle fibers indicated with arrows. **(B)** Each image file was manually processed to isolate myofibers of the transverse muscles, and to remove areas of mechanical artifacts. **(B**^**1**^**)** Zoomed image excerpt from the middle region of the intrinsic tongue shown in panel **B**. **(C)** The Matlab application SMASH was used to identify myofibers, shown here in arbitrary pseudocolor. **(C**^**1**^**)** Zoomed excerpt from the middle region of the intrinsic tongue shown in panel **C**.

In Adobe Photoshop, each intrinsic tongue image was manually divided into three separate regions of equal length to separate anterior, middle, and posterior regions for analysis (Figure 3A). Tongues were separated into these three regions for analysis because it has been reported that anterior, middle, and posterior intrinsic tongue regions differ in their muscle biology and physiology (46, 47). The anterior limit of the tongue was defined as the tongue tip. The posterior limit of the tongue was defined as the region anterior to the sublingual salivary glands, and/or anterior to the transition to smooth mucosa on the dorsal tongue. The middle region of the tongue was defined to contain the frenulum (the region where the limit of ventral tongue mucosa is reached). The distance between the anterior tongue tip and the posterior border of the tongue was measured, and this distance was used to separate the image into three equal sections of the anterior, middle, and posterior regions. This strategy ensured that the relative sizes of each region were scaled appropriately to each individual tongue, while also ensuring reproducible and standardized identification of anatomical regions across all experimental groups. Next, transverse muscle fibers were isolated by manually removing SL, IL, and verticalis fibers of the intrinsic tongue. This resulted in anterior, middle, and posterior images of the tongue that contained only transverse muscle fibers (Figure 3B).

Each regional image of the tongue was analyzed using the MATLAB application SMASH as previously described (48) to quantify the size and MyHC isoform expression of each transverse muscle fiber (Figure 3C). Between several hundred and a few thousand transverse myofibers were analyzed for each region of each tongue (Supplementary Figure S2) to generate a mean value for measures of each tongue region of each mouse. Minimum feret diameter (the shortest distance across a myofiber) and cross-sectional area (CSA) were the measurements used for the size of myofibers (48), the borders of which were identified by the laminin staining. MyHC staining was used to determine the percentages of myofibers that were positive for each MyHC isoform. At the end of SMASH processing, each of the three regions of a tongue (anterior, middle, and posterior) generated a set of six datapoints for each mouse resulting in a total of 18 different myofiber measures for each mouse. These were the average minimum feret diameter, average CSA, percentage of myofibers that were positive for MyHC 2a and their average CSA, and percentage of myofibers that were positive for MyHC 2b and their average CSA, analyzed separately for anterior, middle, and posterior regions of the transverse muscle.

### 2.7 Statistical analysis

#### 2.7.1 Analysis of body weight, eating, and drinking behavior

Body weight, eating, and drinking behavior were analyzed by 3-way ANOVA to evaluate significant effects for genotype, age, and sex, and significant interactions between genotype, age, and sex. Tukey’s post-hoc tests were performed to evaluate differences between subgroups solely when significant interactions between independent variables were present.

#### 2.7.2 Analysis of intrinsic tongue anatomy

Multivariate linear regression models were used to evaluate the impacts of genotype, age, and sex on the intrinsic tongue muscle measures, as well as transverse intrinsic myofiber measures. Further, since weight and age were highly associated and not appropriate to be included in one model, subgroup analysis of each age group was performed with covariates of weight, genotype, and sex. The intrinsic tongue muscle measures evaluated were area and size measurements of SL, IL, and combined areas of transverse and verticalis (T/V) muscles (mm^2^), tongue length (mm), tongue section area (mm^2^), and the relative proportion (%) of tissue section area that was comprised of SL, IL, T/V muscles. Transverse myofiber analysis included six different outcome measures of the transverse intrinsic tongue myofibers (average Minimum Feret fiber diameter, average myofiber cross sectional area (CSA), CSA of MyHC 2a + fibers, CSA of MyHC 2b + fibers), and MyHC measures (% MyHC 2a + fibers, and % MyHC 2b + fibers).

For this study, power analyses of related measures in prior work determined that a target sample size of 13 mice per group, for a total of 156 mice, would be sufficient to detect genotype-specific differences. However, final sample numbers for each experimental measure varied slightly due to factors such as incidental attrition, sporadic technical artifact, or equipment malfunction. Statistical analyses were performed at significance level of 0.05 *a priori*, using software R (version 4.2.1) and GraphPad Prism (version 10.2.3).

## 3 Results

### 3.1 Eating and drinking behavior phenotyping

**Figure 4:**
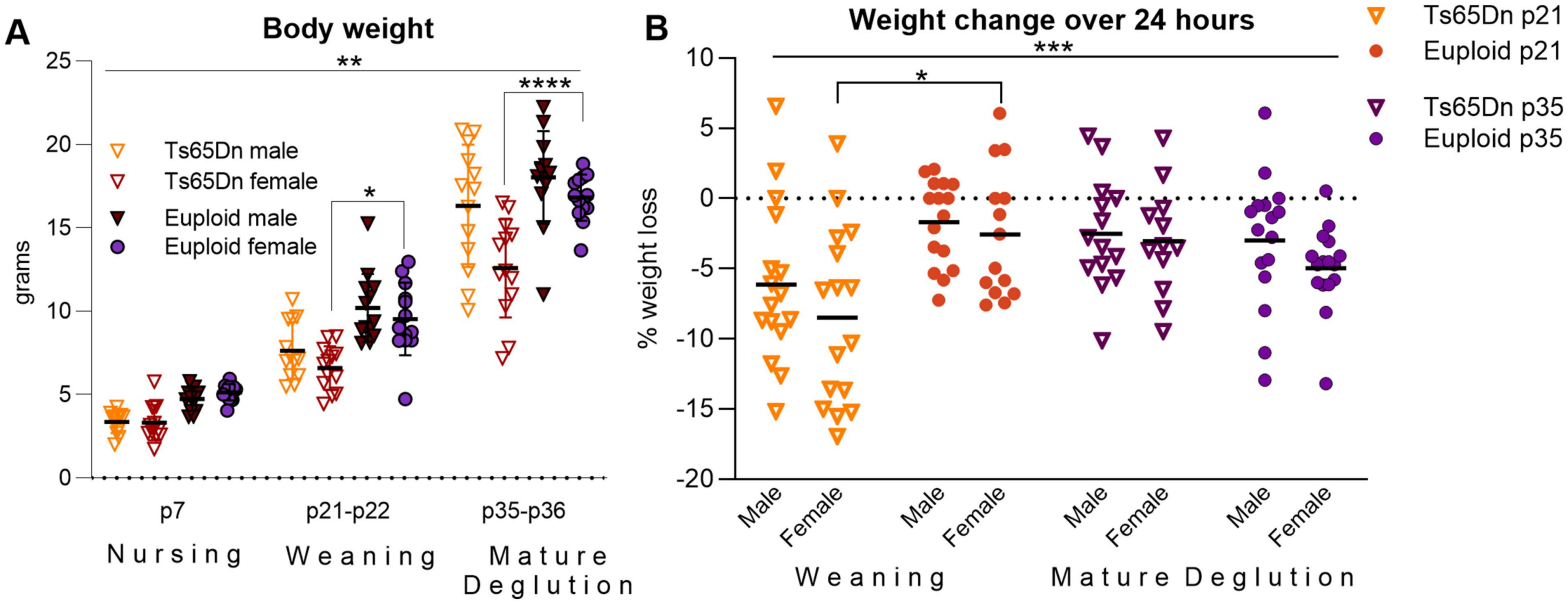
Ts65Dn have smaller body sizes than euploid, and weaning in Ts65Dn is associated with rapid weight loss. **(A)** Ts65Dn are smaller than Euploid, particularly in females at p21 and p35. *n* = 13/group. **(B)** Ts65Dn lose body weight at p21 in a 24-h period at weaning, but do not lose body weight when evaluated over a 24-h period at p35. Ts65Dn p21 males and females *n* = 16/group, Euploid p21 male *n* = 16/group, females 14/group.Ts65Dn p35 male *n* = 14/group, females 12/group. Euploid p35 males and females n = 16/group. Each data point indicates one mouse. Bars show the group mean. * = *p* < 0.05, ** = *p* < 0.01, *** = *p* < 0.001.

**Figure 5:**
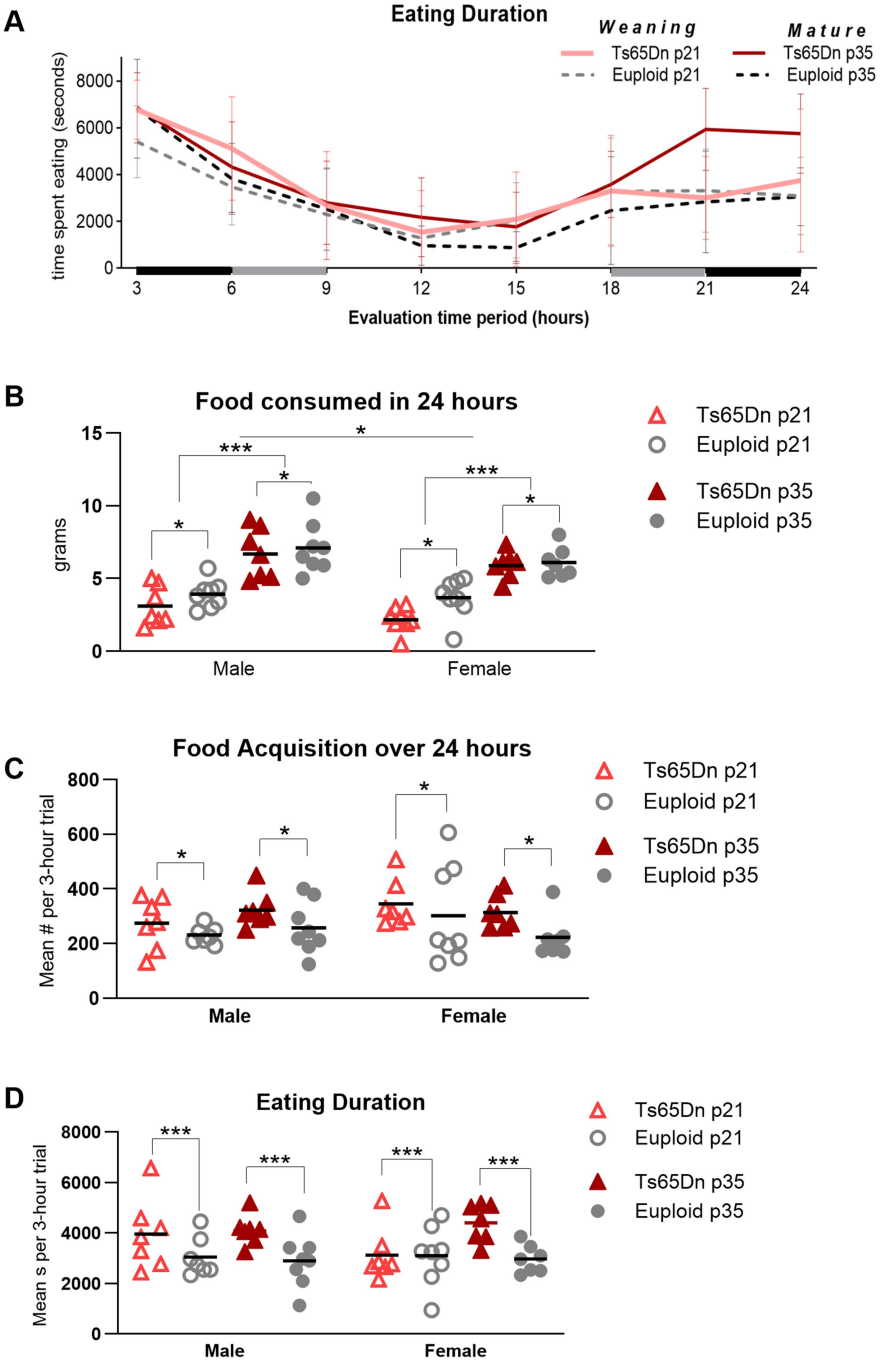
Ts65Dn show feeding phenotypes at p21 (weaning) and p35 (mature deglutition). **(A)** Behavior related to eating and drinking was continuously monitored over 24 h and consolidated into eight consecutive 3-h trials for analysis. Data collection began in the dark cycle, transitioned to 12 h of a light cycle, and ended in the dark cycle. Values for males and females are pooled in this figure panel. Lines and error bars indicate the group means and SD. The x-axis is shaded to indicate the dark cycle (black), and trials that spanned the transition from dark to light cycle (gray). **(B)** Ts65Dn consume less food than euploid controls during a 24-h period at weaning, as well as at p35-36. Mice at p21 consume less food than mice at p35, and female mice consume less food than male mice. **(C)** Ts65Dn access food significantly more often than euploid. **(D)** Ts65Dn spend significantly more time eating than euploid controls. Each data point was generated by two mice of the same age, sex, and genotype. Sample sizes: p21: Ts65Dn M = 14, *F* = 16, p21 Euploid M = 16, *F* = 16. P35: Ts65Dn M = 14, *F* = 14, p35 Euploid M = 16, *F* = 14. * = *p* < 0.05 ** = *p* < 0.01, *** = *p* < 0.001.

Body weight was measured in mice at p7, p21-22, and p35-36 because slow weight gain is frequently regarded clinically as symptom of feeding difficulties in children with DS (49, 50). Prior to p7, mortality in Ts65Dn litters was recorded, and suggested a low to moderate incidence of neonatal death (Supplementary materials S3). In evaluation of body weight, there was a significant interaction between genotype and age [*F* (2, 144) = 5.872, *p* = 0.0035], and a significant independent effect for sex [*F* (1, 144) = 58.47, *p* < 0.0001]. Tukey’s post-hoc analysis indicated significantly lower weights in female Ts65Dn relative to euploid controls at 21–22 (*p* = 0.012) and p35-36 (*p* < 0.0001; Figure 4A). In evaluation of weight changes over a 24-h period at weaning (p21-22) and at p35-36, there was a significant interaction between genotype and age [*F* (1, 112) = 14.49, *p* = 0.0002], in the absence of significant effects for sex, such that Ts65Dn lost weight in the 24 h after weaning when compared to euploid sibling controls. Post-hoc tests additionally indicated significantly lower weight in Ts65Dn females than euploid females at p21-p22 (*p* = 0.013; Figure 4B). Eating and drinking behaviors were quantified over 24 h at p21-22 and p35-36 (Figure 5A). While all experimental groups showed high levels of eating engagement at the initiation of the 24-h assay period, which may have been partly attributable to the transient novelty of the evaluation environment, there were significant differences between genotype groups in some measures of eating behavior.

There were significant differences due to genotype, age, and sex, in the absence of significant interactions between these factors, in the amount of food consumed over the 24-h evaluation period. Ts65Dn consumed significantly less food than euploid [*F* (1, 52) = 5.007, *p* = 0.03], female mice consumed significantly less food than male mice [*F* (1, 52) = 4.995, *p* = 0.03], and p21-22 mice consumed significantly less food than p35-36 mice [*F* (1, 52) = 94.47, *p* < 0.0001] (Figure 5B). In the measure of food acquisition behavior, or the number of times mice accessed the food, there was a significant effect for genotype, in the absence of significant effects for other factors and in the absence of significant interactions between other factors. Ts65Dn accessed food more frequently than euploid [*F* (1, 50) = 5.580, *p* = 0.022; Figure 5C]. In the measure of eating duration, or the amount of time mice spent eating, there was a significant effect for genotype, in the absence of significant effects for other factors and in the absence of significant interactions between other factors. Ts65Dn spent significantly more time eating food during the 24-h period than euploid [*F* (1, 50) = 12.60, *p* = 0.0009; Figure 5D]. Collectively, these data suggest that Ts65Dn differs from euploid in efficacy of eating at both p21 and p35. Ts65Dn ate less food than euploid, but accessed the food significantly more frequently than euploid, and spent significantly more time eating than euploid.

**Figure 6:**
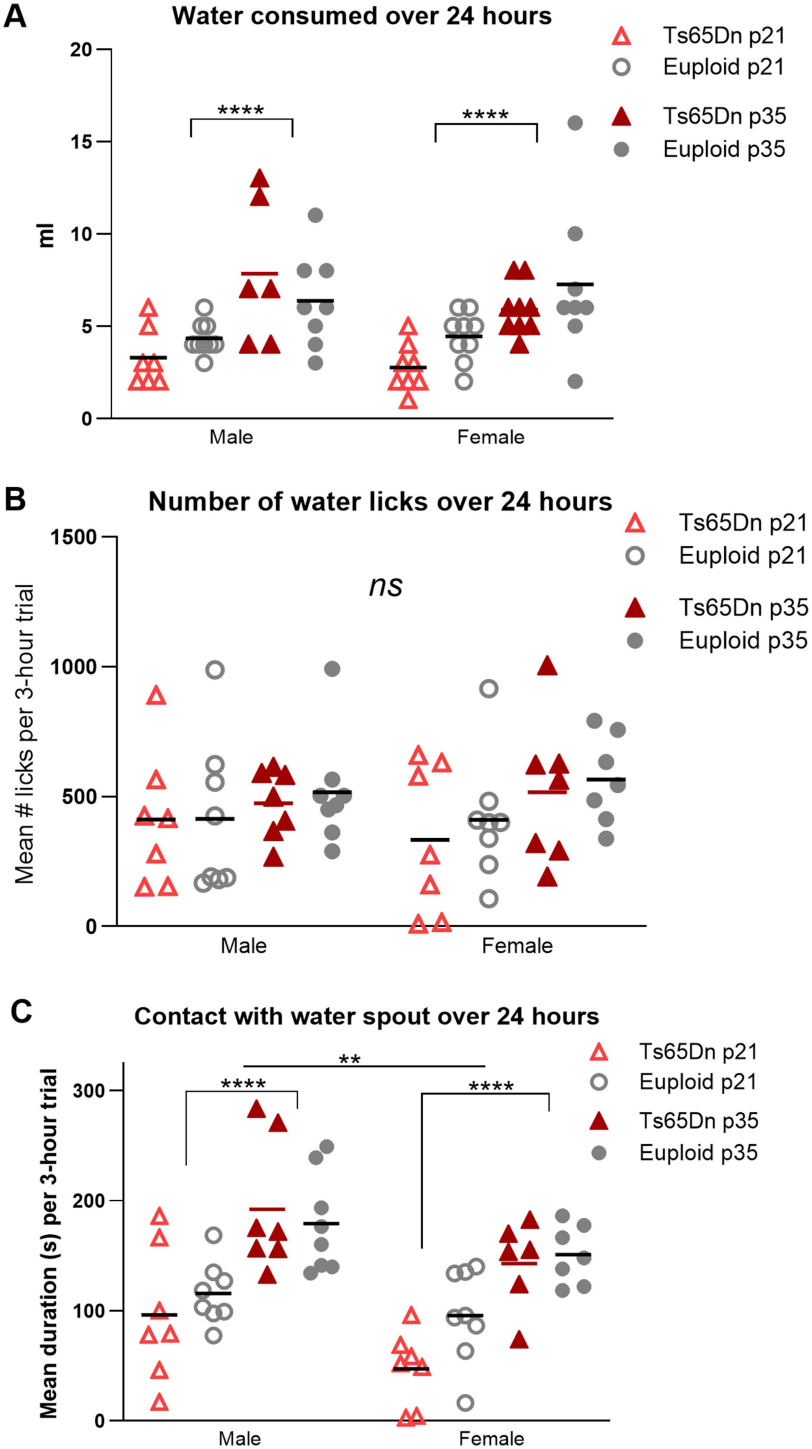
Drinking behavior at p21 (weaning) and p35 (mature deglutition). Each data point was generated by two mice of the same age, sex, and genotype. **(A)** p21 mice consume less water than p35 mice controls during a 24-h period. **(B)** There are no significant differences between groups in the number of water licks during drinking over a 24-h period. **(C)** p21 mice spend less time in contact with the water spout than p35 mice, and female mice spend less time in contact with the water spout than male mice. Bars indicate the group means. Sample sizes: p21: Ts65Dn M = 14, F = 16, p21 Euploid M = 18, *F* = 18. P35: Ts65Dn M = 18, F = 18, p35 Euploid M = 16, F = 16. ** = *p* < 0.01, **** = *p* < 0.0001.

In the amount of water consumed, there was a significant effect for age in the absence of significant interactions between age, genotype, and sex, such that p35-36 mice consumed more water than p21-22 mice [*F* (1, 56) = 28.41, *p* < 0.0001]. While group means of water consumed by Ts65Dn at p21 were lower than euploid at p21, these differences did not achieve statistical significance (Figure 6A). There were no differences between groups in the number of licks used for drinking during the evaluation period (Figure 6B). In evaluation of the amount of time mice spent in physical contact with the water spout, there were significant effects for age and sex, in the absence of significant effects for genotype or significant interactions between age, sex, and genotype. Mice spent less time in contact with the water spout at p21 compared to p35 [*F* (1,50) = 45.81, *p* < 0.0001], and female mice spent less time in contact with the water spout than male mice [*F* (1,50) = 10.21, *p* = 0.002]. While group means of contact time for Ts65Dn were below those of euploid mice, these differences did not attain statistical significance (Figure 6C). While it is plausible that some of these measures may relate to mouse body size, evaluation of eating and drinking measures for significant correlations with individual mouse body weight were not performed because these behavioral analyses were conducted on pairs of mice, such that the accuracy of detection of relationships between individual body weight and paired eating and drinking measures was intrinsically limited for this experimental paradigm.

### 3.2 Intrinsic tongue muscle and myofiber analysis

**Figure 7:**
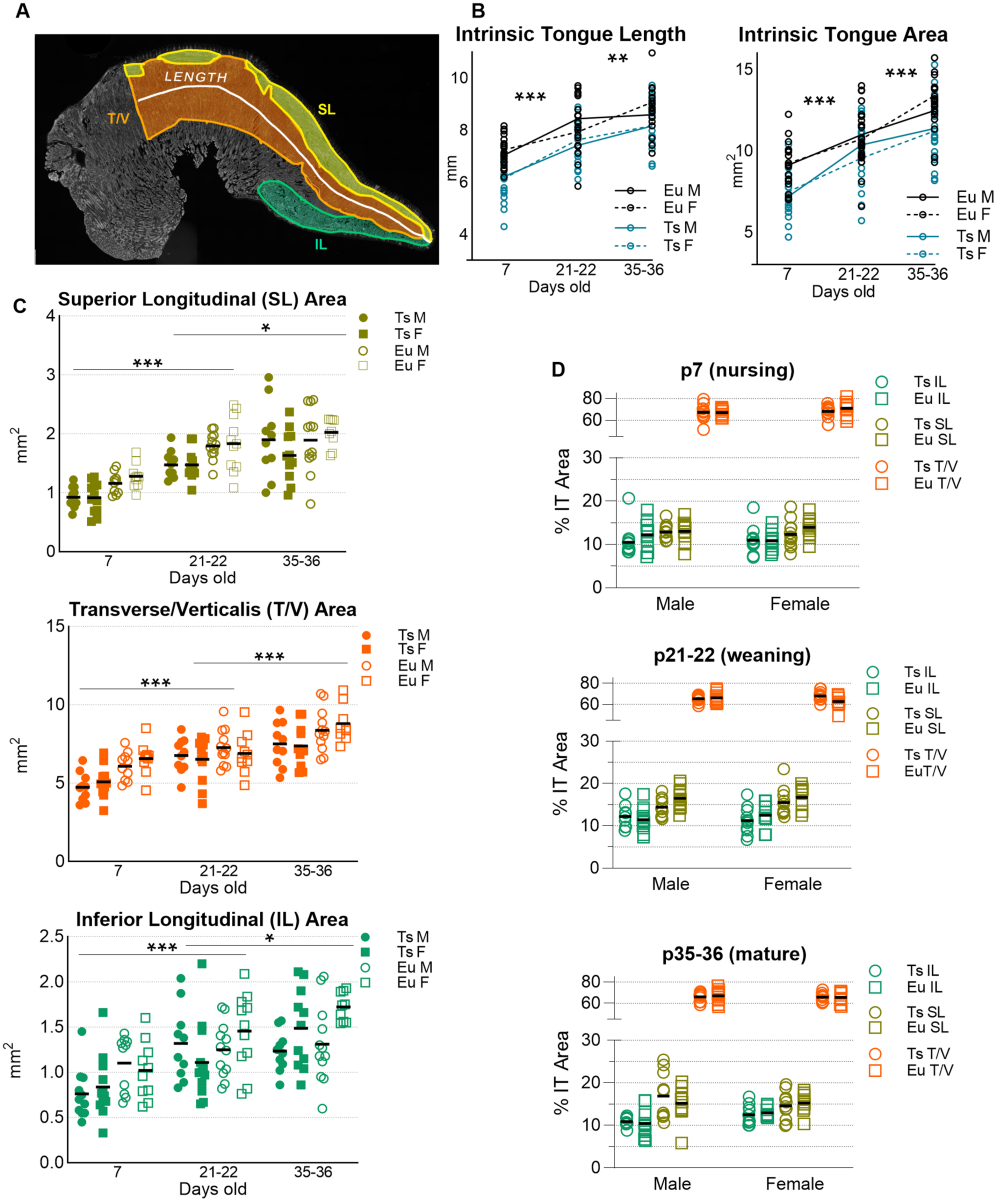
Sizes of Intrinsic Tongue (IT) muscles during postnatal development. **(A)** superior longitudinal (SL), Inferior longitudinal (IL), Transverse / Verticalis (T/V) muscles, IT length, and total IT area were manually measured in one tissue section from each mouse. Three-color microscopy images stained for MyHC 2a, 2b, and laminin were analyzed in color, but this example is shown in grayscale for clarity to illustrate the locations of the IT muscles. **(B)** Ts65Dn showed smaller values for IT length and IT area than euploid. **(C)** SL, T/V, and IL muscles are shown at each age point analyzed. **(D)** IT muscle sizes are shown normalized to total IT area. Each data point indicates analysis from one tissue section of one mouse. P7 sample sizes: Ts M = 11, Ts *F* = 12, Eu M = 10, Eu *F* = 10. P21 sample sizes: Ts M = 10, Ts F = 12, Eu M = 12, Eu *F* = 11. P35 sample sizes: Ts M = 10, Ts F = 11, Eu M = 12, Eu F = 10. Ts = Ts65Dn, Eu = Euploid, M = male, F = female. Bars show the mean. * = *p* < 0.05, ** = *p* < 0.01, *** = *p* < 0.001.

**Figure 8:**
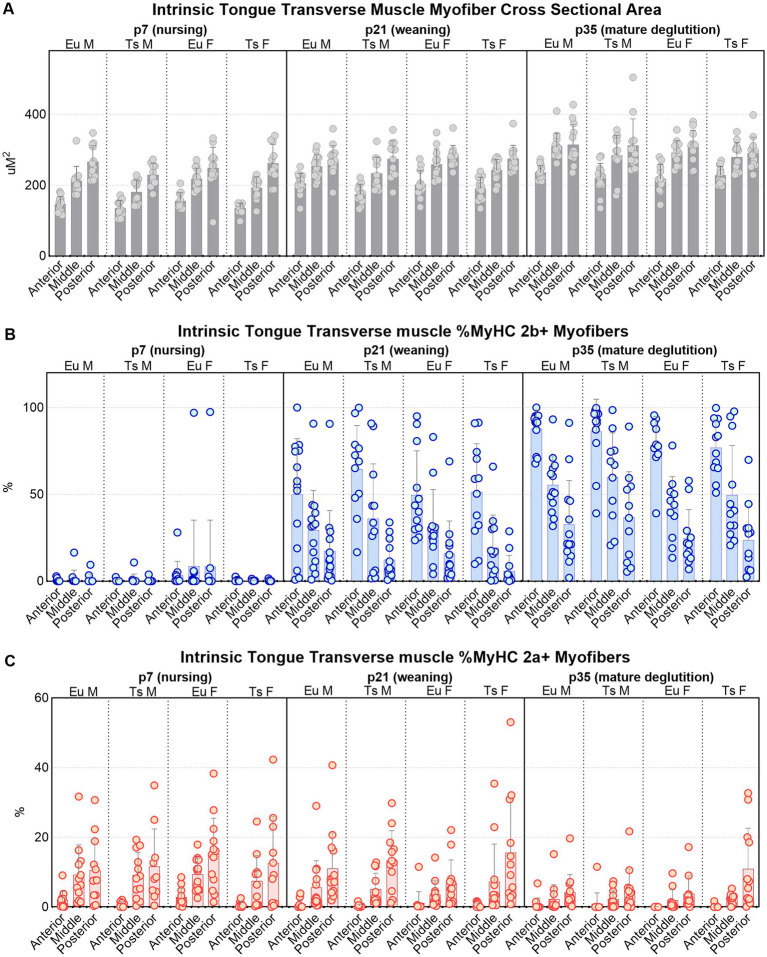
Quantification of myofiber measures in the anterior, middle, and posterior regions of the intrinsic tongue transverse muscle in Ts65Dn and euploid controls. **(A)** Cross-sectional area (CSA) is significantly different with genotype, age, and anatomical region. **(B)** The percentage of myofibers that express MyHC 2b are significantly different with age and anatomical region. **(C)** The percentage of myofibers that express MyHC 2a are significantly different with genotype, age, and anatomical region. Each data point indicates the mean value of myofibers from one tissue section of one mouse. Bars indicate the group mean and SD. Eu = euploid, Ts = Ts65Dn, M = male, F = female. Sample sizes: p7: Ts M = 12, Ts F = 12, Eu M = 12, Eu *F* = 13. p21: Ts M = 12, Ts F = 12, Eu M = 14, Eu *F* = 12. p35: Ts M = 13, Ts F = 12, Eu M = 13, Eu F = 11.

Tissue staining and microscopy generated a dataset of 154 high-resolution images of intrinsic tongue muscle sections of male and female mice of both genotypes at the three ages studied (p7, p21-22, and p35-36). Tissue section images were analyzed for measures of the overall size of the intrinsic tongue, as well as for the area of each of the four major intrinsic tongue muscles (Figure 7). Of the four major intrinsic tongue muscles, the T/V muscles were selected for expanded analysis of myofiber characteristics (Figure 8).

#### 3.2.1 Intrinsic tongue muscle analysis

Intrinsic tongue muscle sizes increased significantly with age, such that p7 tongues were shorter than p21 tongues (*p* < 0.001), and p21 tongues were shorter than p35 tongues (*p* = 0.004). Similarly, tongue tissue section areas were smaller in p7 mice than p21 mice (*p* < 0.001), and were also smaller in p21 mice than p35 mice (*p* < 0.001). Sex-specific differences were not detected. Compared to euploid, Ts65Dn genotype also resulted in significantly smaller values for all measures of intrinsic tongue muscle size such that Ts65Dn tongues were smaller than euploid tongues in measures of tongue section length (*p* < 0.001) and tongue section area (*p* < 0.001). However, because the Ts65Dn genotype can coincide with smaller overal body sizes than euploid at young ages, these measures were also analyzed with body weight as a co-variate to consider the possibility that tongue size differences in Ts65Dn are attributable to overall smaller body sizes. When analyzed with body weight as a covariate, genotype did not confer significant differences in these measures of tongue muscle size. While final sample sizes in this subgroup analysis were slightly less than the study target sample sizes, raising the possibility that subgroup analysis was slightly underpowered for the detection of genotype-specific differences, this outcome is also compatible with the possibility that tongue size differences between Ts65Dn and euploid may be at least partly attributable to genotype-specific differences in overall mouse size (Figures 7A,B).

Similarly, in analysis of the different intrinsic tongue muscles, muscle size increased significantly with age, in that between p7 and p21 there were significant increases in the area sizes of the SL (*p* < 0.001), T/V (*p* < 0.001), and IL (*p* < 0.001). Age was also associated with greater size from p21 to p35 of the SL (*p* = 0.011), T/V (*p* < 0.001), and IL (*p* = 0.037). Sex-specific differences in these measures were not detected. Upon intitial analysis, Ts65Dn had smaller section areas than euploid of the SL (*p* < 0.001), T/V (*p* < 0.001), and IL (*p* = 0.002) muscles. However, when these measures were analyzed with weight as a co-variate, no significant genotype-specific differences were detected. This is compatible with a possibility that size differences of different intrinsic tongue muscles between Ts65Dn and euploid may be at least partly attributable to genotype-specific differences in overall mouse size (Figure 7C).

Because there was a correlation between body weight and the physical size of intrinsic tongue muscles, intrinsic tongue muscle size data were also separately analyzed as a percentage of the overall intrinsic tongue section size, thereby normalizing the areas of each intrinsic tongue muscle to the total intrinsic tongue section size for each mouse. When evaluated through this alternative approach to controlling for body size, neither genotype-specific nor sex-specific differences in the relative IT muscle sizes were detected (Figure 7D).

#### 3.2.2 Transverse tongue myofiber analysis

**Figure 9:**
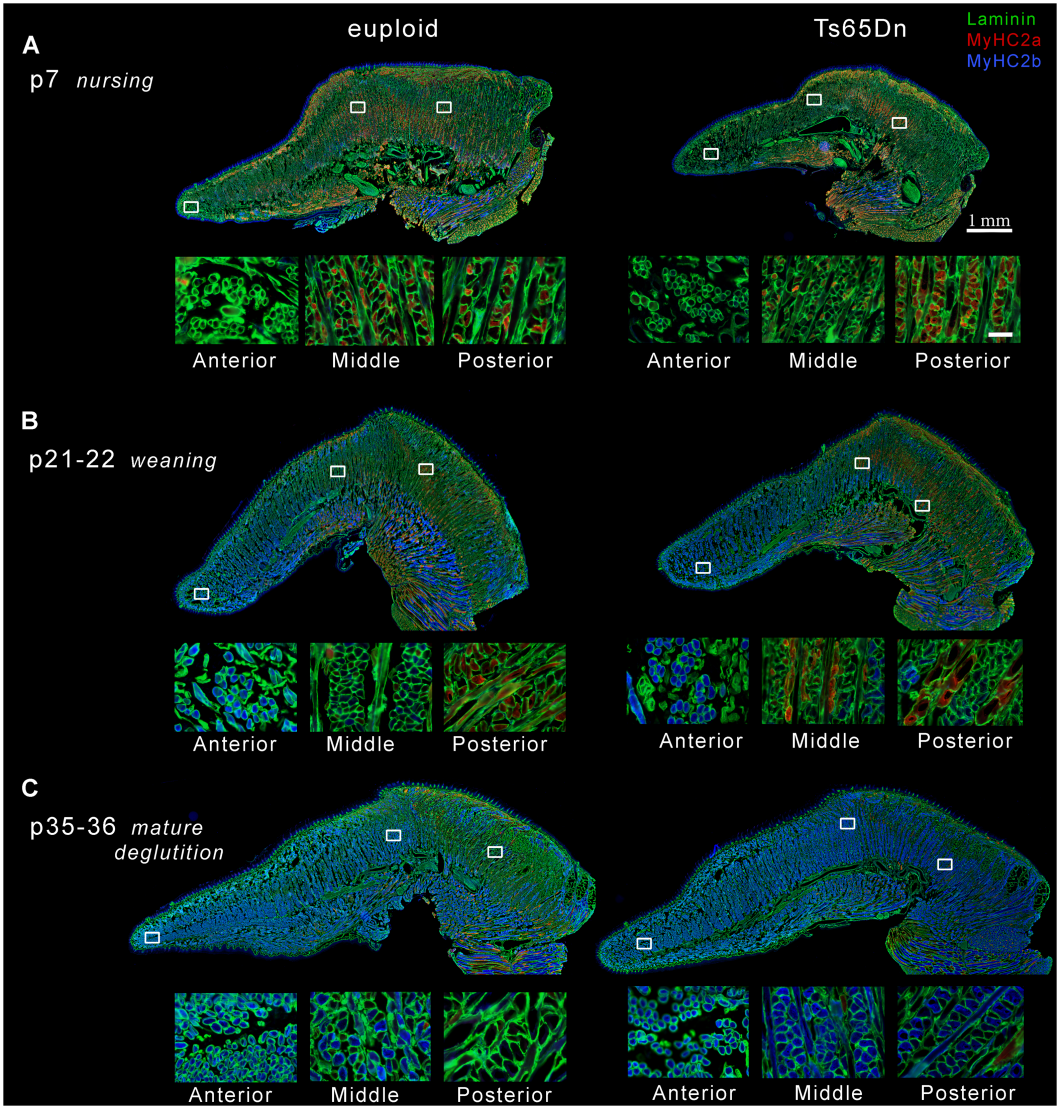
Representative images of intrinsic tongues of Ts65Dn and euploid controls. In each panel, one entire tissue section is shown above (scale bar indicates 1 mm), and enlarged excerpts from the locations indicated by white boxes are shown below (scale bar indicates 50 μm). **(A)** Intrinsic tongue sections from p7 pups reveal predominance of MyHC 2a staining primarily in the posterior regions, and shows smaller myofibers in the anterior regions. **(B)** Intrinsic tongue sections from p21-22 pups reveal onset of MyHC 2b staining in the anterior tongue at this age, retention of MyHC 2a staining in posterior tongue, and smaller myofibers in the anterior tongue regions. **(C)** Intrinsic tongue sections from p35-36 mice reveal expansion of MyHC 2b staining to middle and posterior regions, relative loss of MyHC 2a, and smaller myofibers in the anterior tongue regions. For clarity, the intensity of red and blue signal has been doubled relative to green signal in each intrinsic tongue section micrograph in the top panels of **A–C**.

Semi-automatic muscle analysis using segmentataion of histology (SMASH) of the transverse muscle myofibers of intrinsic tongue indicated considerable differences as a function of anatomical region of the intrinsic tongue (anterior, middle, or posterior), as well as by age (p7; nursing, p21-22; weaning, p35-36; mature deglutition), as well as by genotype (Ts65Dn vs. euploid). There were no significant differences between males and females in any of the measures of transverse myofiber properties (Figures 8, 9; Table 1).

**Table 1:** Summary table of multivariate analysis of myofibers of the transverse intrinsic tongue muscle.

Intrinsic tongue region	Myofiber measure	Characteristic	*β*	95% CI	*p* value
Anterior transverse muscle	Minimum feret	Genotype (Ts65Dn vs. euploid)	−0.40	−0.74, −0.05	0.025*
		Age (p7 vs. p21)	−1.7	−2.2, −1.3	<0.001***
		Age (p21 vs. p35)	0.98	0.56, 1.4	<0.001***
	CSA	Genotype (Ts65Dn vs. euploid)	−13	−22, −3.3	0.009**
		Age (p7 vs. p21)	−51	−63, −39	<0.001***
		Age (p21 vs. p35)	32	21, 44	<0.001***
	% MyHC 2b+	Genotype (Ts65Dn vs. euploid)	−0.21	−6.9, 6.5	0.950
		Age (p7 vs. p21)	−51	−60, −43	<0.001***
		Age (p21 vs. p35)	28	20, 36	<0.001***
	MyHC 2b + CSA	Genotype (Ts65Dn vs. euploid)	−28	−46, −11	0.002***
		Age (p7 vs. p21)	−61	−84, −38	<0.001***
		Age (p21 vs. p35)	39	19, 60	<0.001***
	% MyHC 2a+	Genotype (Ts65Dn vs. euploid)	−0.70	−1.3, −0.07	0.029*
		Age (p7 vs. p21)	0.90	0.13, 1.7	0.022*
		Age (p21 vs. p35)	−0.17	−0.93, 0.60	0.668
	MyHC 2a + CSA	Genotype (Ts65Dn vs. euploid)	7.8	−14, 30	0.482
		Age (p7 vs. p21)	−48	−72, −24	<0.001***
		Age (p21 vs. p35)	25	−6.9, 57	0.123
Middle transverse muscle	Minimum feret	Genotype (Ts65Dn vs. euploid)	−0.75	−1.2, −0.34	<0.001***
		Age (p7 vs. p21)	−1.5	−2.0, −0.96	<0.001***
		Age (p21 vs. p35)	1.4	0.91, 1.9	<0.001***
	CSA	Genotype (Ts65Dn vs. euploid)	−22	−34, −9.7	<0.001***
		Age (p7 vs. p21)	−45	−60, −30	<0.001***
		Age (p21 vs. p35)	45	29, 60	<0.001***
	% MyHC 2b+	Genotype (Ts65Dn vs. euploid)	−1.6	−8.6, 5.3	0.645
		Age (p7 vs. p21)	−25	−34, −17	<0.001***
		Age (p21 vs. p35)	22	14, 31	<0.001***
	MyHC 2b + CSA	Genotype (Ts65Dn vs. euploid)	−36	−59, −14	0.002***
		Age (p7 vs. p21)	−79	−109, −50	<0.001***
		Age (p21 vs. p35)	44	19, 69	<0.001***
	% MyHC 2a+	Genotype (Ts65Dn vs. euploid)	0.30	−1.7, 2.3	0.760
		Age (p7 vs. p21)	3.3	0.95, 5.7	0.007**
		Age (p21 vs. p35)	−3.2	−5.6, −0.78	0.010**
	MyHC 2a + CSA	Genotype (Ts65Dn vs. euploid)	10	−3.0, 23	0.129
		Age (p7 vs. p21)	−35	−50, −19	<0.001***
		Age (p21 vs. p35)	18	2.2, 34	0.027*
Posterior transverse muscle	Minimum feret	Genotype (Ts65Dn vs. euploid)	−0.14	−0.62, 0.34	0.574
		Age (p7 vs. p21)	−0.82	−1.4, −0.23	0.007*
		Age (p21 vs. p35)	0.85	0.27, 1.4	0.004**
	CSA	Genotype (Ts65Dn vs. euploid)	−6.2	−22, 9.8	0.443
		Age (p7 vs. p21)	−25	−44, −5.0	0.014*
		Age (p21 vs. p35)	30	11, 50	0.002**
	% MyHC 2b+	Genotype (Ts65Dn vs. euploid)	−4.3	−10, 1.8	0.168
		Age (p7 vs. p21)	−9.9	−17, −2.3	0.011**
		Age (p21 vs. p35)	16	8.7, 23	<0.001***
	MyHC 2b + CSA	Genotype (Ts65Dn vs. euploid)	−0.78	−34, 32	0.963
		Age (p7 vs. p21)	−126	−176, −76	<0.001***
		Age (p21 vs. p35)	14	−22, 49	0.445
	% MyHC 2a+	Genotype (Ts65Dn vs. euploid)	2.0	−1.0, 5.1	0.195
		Age (p7 vs. p21)	2.0	−1.7, 5.8	0.290
		Age (p21 vs. p35)	−4.6	−8.3, −0.86	0.016**
	MyHC 2a + CSA	Genotype (Ts65Dn vs. euploid)	8.7	−6.9, 24	0.272
		Age (p7 vs. p21)	−37	−56, −18	<0.001***
		Age (p21 vs. p35)	44	25, 63	<0.001***

A multivariate linear regression model was used to evaluate transverse intrinsic muscle myofiber measures obtained from SMASH analysis. Values were compared to the reference age of p21 and the reference genotype of euploid. CI = Confidence interval. CSA = myofiber cross-sectional area. For all measures there were no significant differences between sexes (male vs. female). While the impacts of weight and sex are not reflected in Table 1; Supplementary Tables S1–S3 contain results of subgroup analysis that was performed with weight, genotype and sex as covariates.

Accross all three ages, the anterior region of the transverse muscle was comprised of the smallest myofibers of the three regions, while myofibers in the middle region were larger, and myofibers in the posterior region were the largest (Figures 8A, 9). Additionally, myofiber sizes of the transverse muscle were smallest at p7, larger at p21-22, and largest at p35-36. All measures of muscle fiber size in all of the three regions of the intrinsic tongue were significantly different with age, such that values for the myofiber size measures of minimum feret and CSA were significantly smaller in younger mice and larger in older mice (Figure 8A; Table 1). Multivariate analysis suggested that compared to euploid, the Ts65Dn genotype may confer anatomically specific differences in myofiber size measures in specific regions of the intrinsic tongue, such that the anterior region and the middle region of the transverse muscle were impacted by genotype, but the posterior region of the intrinsic tongue transverse muscle was unaffected by genotype. These differences included smaller CSA and smaller minimum feret diameter in the anterior transverse muscle and middle transverse muscle in Ts65Dn as compared to euploid, but no differences between genotypes in myofiber sizes of the posterior transverse muscle (Table 1). This indicates that while mice of both genotypes have an anterior to posterior gradient in myofiber sizes, Ts65Dn tongues have larger posterior myofibers relative to the smaller sizes of the anterior and middle myofibers of the transverse muscle. Quantification of the percentage of myofibers expressing MyHC 2b, a marker for very fast, fatiguable muscle fibers, showed differences as a function of anatomical region (anterior, middle, posterior) and age (p7, p21-22, p35-36). There was a strong relationship between MyHC 2b expression and age, such that the lowest expression of MyHC 2b occurred during p7 (nursing), greater amounts of MyHC 2b were expressed at p21-22 (weaning), and the greatest proportion of fibers expressing MyHC 2b occurred at p35-36 (mature deglutition). While p7 tongues did not express appreciable amounts of MyHC 2b, at p21-22 and p35-36 the greatest percentage of myofibers expressed MyHC 2b in anterior tongue regions, a smaller percentage of myofibers expressed MyHC 2b in middle tongue regions, and the smallest percentage of myofibers expressed MyHC 2b in posterior tongue regions. Ts65Dn showed significantly smaller CSA of MyHC 2b + fibers specifically in the anterior and middle regions of the transverse muscle, but not in the posterior regions. However, Ts65Dn and euploid had similar proportions of myofibers that expressed MyHC 2b (Figures 8B, 9; Table 1).

The proportion of myofibers expressing MyHC 2a, a marker for moderately fast muscle fibers, showed significant differences as a function of anatomical region as well as age and genotype. Across all groups, the lowest percentage of myofibers expressed MyHC 2a in anterior tongue, a larger percentage of myofibers expressed MyHC 2a in middle tongue, and the largest percentage of myofibers expressed MyHC 2a in posterior tongue. Additionally, there was a strong relationship between MyHC 2a expression and age, such that the highest expression of MyHC 2a occurred during p7 (nursing), lower amounts of MyHC 2a were expressed at p21-22 (weaning), and the lowest proportion of fibers expressing MyHC 2a occurred at p35-36 (mature deglutition). Ts65Dn had significantly lower percentages of myofibers positive for MyHC 2a specifically in the anterior region of the transverse tongue muscle (Figures 8C, 9; Table 1).

Finally, to accommodate the possibility that differences in overall mouse size could be implicated in genotype-specific differences in tongue myofiber sizes, all transverse muscle myofiber data were also separately evaluated in subgroup analysis at each age, with bodyweight, sex, and genotype as co-variates (Supplementary Tables S1–S3). When analyzed with co-variates which controled for differences attributable to animal size, many of the genotype-specific differences in myofiber size detected in Ts65Dn were determined not to be significant. This is compatible with the interpretation that myofiber sizes of anterior and middle intrinsic tongue regions in Ts65Dn may be generally proportional to the smaller body sizes of Ts65Dn. However, when intrinsic tongue myofiber data was analyzed with co-variates, Ts65Dn were found to have significantly larger MyHC 2a + fibers than euploid specifically in the anterior region of the transverse muscle at p7, as well as at p35-56. This is compatible with the interpretation that Ts65Dn may have MyHC 2a + fiber-specific differences in specific tongue regions at specific ages that are not explained by body size alone. In summary, Ts65Dn appear to have differences in tongue myofiber sizes that may be region-specific and partially explained by differences in overall body size, and since two different analysis strategies indicated genotype-specific differences in MyHC 2a + fibers in Ts65Dn intrinsic tongue muscles, it is possible to conclude that age-specific differences specific to certain types of myofibers occur in the tongue of this mouse model of DS.

## 4 Discussion

This study used the Ts65Dn mouse model of DS and sibling controls to test the hypothesis that DS is associated with developmental delays in maturation of the tongue muscle system. Functional oromotor measures and intrinsic tongue muscles were analyzed at three postnatal developmental timepoints representative of nursing (p7), weaning (p21), and mature deglutition (p35) (51). These three ages were chosen because each of them differs in how the intrinsic tongue muscles facilitate oral intake. Some of our findings were broadly compatible with the hypothesis of developmental delay, while other findings were more indicative of developmental differences rather than developmental delays.

Body weight, used to indicate developmental challenges with deglutition in clinical contexts (52), was lower in Ts65Dn than euploid mice, and also showed significant differences in Ts65Dn specifically at weaning. Ts65Dn showed significant differences from euploid in weight change and eating measures at p21, as well as in eating measures at p35. Compared to euploid, Ts65Dn also showed differences in intrinsic tongue muscle microanatomy and biology at p7, p21 and p35. These anatomical differences included smaller myofiber sizes in anterior and middle transverse intrinsic tongue regions, but not in posterior transverse intrinsic tongue regions, and lower proportions of MyHC2a + myofibers specifically in the anterior transverse muscle. Further, MyHC2a + myofibers in the anterior transverse muscle were found to be relatively larger in Ts65Dn at p7 and p35 when evaluated with body weight as a covariate. Collectively this suggests that while Ts65Dn may have some regional myofiber differences in anterior and middle tongue regions that are partially attributable to genotype-specific differences in overall animal size, Ts65Dn may also have myofiber differences that are not explained by differences in animal size. In sum, while there are significant genotype-specific differences in intrinsic tongue muscles and in behaviors dependent on the tongue muscle system, the framework of developmental delay is not entirely applicable and some of these phenotypes may be more appropriately referred to as developmental differences rather than delays.

Ts65Dn shows significant and rapid weight loss compared to euploid controls in the 24 h after weaning. During this time, despite the fact that Ts65Dn spent more time in food acquisition behavior, and spent more time eating than euploid, Ts65Dn ingested significantly less food than euploid. This weight loss in Ts65Dn may be compatible with an interpretation of developmental delay related to weaning, because weaning entails a dramatic advance in oromotor skills required for food and water intake, and the Ts65Dn phenotype of weight loss is not present at the later timepoint of p35. Prior studies have considered a variety of factors that may contribute to differences in weight gain following weaning in typically developing mice (53, 54). The switch from maternal milk to other food at weaning causes a decrease in body fat percentage, however, substantial weight loss at weaning is not typical for mice; even for mice that have been preemptively selected for smaller body size. The fact that the eating and drinking abilities required for successful weight gain after weaning are developmentally specific is indicated by prior work showing that when typical mice are weaned a week early; at p14, they are unable to gain any weight for the next several days (34). Although it is often believed to be necessary to wean mice at p21 to comply with laboratory husbandry logistics, it is likely that many mice would benefit from weaning at later ages (51), and p21 may be an early weaning date that is particularly challenging for Ts65Dn. Many children with DS have exceptional delays in weaning and delays in developmental transitions to expanded diets, such that they may require special accommodations for eating and drinking during childhood (55, 56).

This study generated comprehensive normative data characterizing the postnatal maturation of the intrinsic tongue muscles in mice, with greater and more comprehensive microanatomical description than has been previously available. Findings of very strong anterior-to-posterior gradients in myofiber biology of the tongue are broadly congruent with prior anatomical studies of adult rat tongues, which demonstrated similar anatomical specificity of MyHC expression in intrinsic tongue muscles (24, 46). Further, our findings of dramatic age-specific changes in MyHC expression are broadly congruent with a prior study reporting significant alterations of MyHC gene expression in the intrinsic tongue that are dependent on weaning. In both cases, MyHC 2b has been found to upregulate at weaning, and MyHC 2a has been found to downregulate at weaning (26). Findings in the current study of differences in MyHC 2a + tongue myofibers fibers in Ts65Dn are interesting in light of a prior report of significant differences in tongue muscle MyHC 2a expression that occur as a result of delays in weaning in a different mouse model of a developmental disorder involving an absence of tooth eruption (27).

An unexpected finding of this study was that some phenotypes of the intrinsic tongue muscles in Ts65Dn were isolated to the anterior and middle regions of the intrinsic tongue muscles but were not detected in the posterior tongue muscles. Myofiber sizes were smaller in Ts65Dn only in the anterior and middle transverse tongue regions but were not smaller than euploid in the posterior intrinsic tongue region. That is, the posterior transverse tongue myofibers of Ts65Dn are disproportionately large relative to the anterior and middle transverse tongue myofibers. This may be of translational interest because the posterior tongue or tongue base is often implicated in upper airway obstruction in children with DS, and anterior-to-posterior collapse of the posterior tongue can pose an otolaryngological challenge (57). Another finding of this study was that the anterior tongue regions of Ts65Dn showed significant differences of MyHC 2a expression compared to euploid. This may be of translational interest because different MyHC isoforms are associated with different muscle contractile properties, and different regions of the intrinsic tongue muscles likely have different roles in deglutition. For example, the anterior tongue is the initial point of contact for water during licking, while the posterior tongue is likely more critical for management of the intraoral bolus. Strength and endurance of anterior vs. posterior regions of the tongue have been topics of pre-clinical study in the context of feeding challenges (58, 59). Because Ts65Dn showed significant weight loss at the timepoint of weaning, which is a process known to be frequently delayed or challenging for many children with DS (55), and also spent longer amounts of time eating relative to the amount of food being consumed, Ts65Dn appears to demonstrate a variety of phenotypes applicable to further study of developmental deglutition challenges in DS that involve tongue function.

While this study provides a more comprehensive examination of tongue muscle maturation phenotypes associated with a mouse model of DS than has been previously available, there were also some limitations in its approach. First, anatomical aspects of intrinsic tongue maturation are likely influenced by skeletal constraints of the maxilla and mandible, which are known to be unique in Ts65Dn (40, 60). Challenges related to tongue size in DS are often attributed to relative macroglossia, or the relationship between the tongue size and the size of the oral cavity (40, 61). Therefore, it is impossible to identify a particular tongue size as problematic without considering the size of the oral cavity in which the tongue is located. While overall measures of tongue size in this study were significantly smaller in Ts65Dn than euploids, and it appears plausible that this may be at least partially explained by smaller overall body size of Ts65Dn, anatomical analysis of the greater oral cavity was not included in this study design and therefore it is not possible to determine whether the tongue size measures in this study reflect either the presence or absence of relative macroglossia. However, a prior study has reported that youth with DS do have significantly smaller tongues compared to youth without DS, and that further, the size of the tongues of youth with DS are still proportionately larger than controls when evaluated relative to craniofacial measurements (62). Future work to expand in this area may be translationally applicable in light of the fact that some genotype-specific myofiber findings occurred in particular regions of the tongue muscle. Because DS can entail challenges specific to the relative size of posterior tongue regions (4), it is translationally interesting that Ts65Dn appeared to demonstrate smaller myofibers in the anterior and middle tongue regions, but not posterior tongue regions.

Secondly, given the 3D complexity of the intrinsic tongue muscles, some measures of the 2D tissue section analysis strategy used here to estimate intrinsic tongue muscle sizes were likely vulnerable to variability caused by small variations in sectioning plane. Even small medial-to-lateral variations in section plane could have impacts on the relative proportions of the different intrinsic tongue muscles represented in each section. It is plausible that the estimation of the sizes of the IL muscles in particular may have been affected by this issue, and therefore findings related to the IL muscle in this study may be appropriately regarded as exploratory. Limitations intrinsic to 2D analysis of tissue sections may also be applicable to transverse myofiber measures in this study. Transverse myofibers were identified through anatomical location and fiber orientation, however, myofiber orientations of intrinsic tongue muscles are very complex and are most accurately appreciated in 3D. 2D analysis may entail risks that myofibers that could have been identified as belonging to the verticalis muscle when evaluated in 3D may have occasionally been identified as belonging to the transverse muscle when evaluated in 2D sections. Future work in 3D imaging to characterize the anatomical changes in the major intrinsic tongue muscles with maturation and in the context of developmental disorders would be of benefit to build on and expand these initial findings.

A third limitation in this study pertains to the biological interpretation of weight loss in Ts65Dn at weaning. While weight loss or slow weight gain can be a sign of pediatric feeding challenges, it is possible that body weight could also be impacted by other phenotypes that may occur in Ts65Dn which were beyond the scope of this study. Those phenotypes include differences in locomotor activity levels and metabolism (63). Because the present study did not quantify physical activity levels or metabolic measures, those factors cannot be ruled out as contributors to weight loss in Ts65Dn at weaning.

As we enter a new era of biomedical research that more equitably includes people with DS and other developmental disorders (64), there is a pressing need to elucidate the biological basis of medical challenges associated with this syndrome. This study provides new information about the postnatal maturation of the intrinsic tongue muscle system, which is critical for several cranial functions that can be impacted in DS. Tongue muscle and myofiber findings in this study clarify aspects of intrinsic tongue development across three anatomical regions of the intrinsic tongue at three functionally distinct time points, within two genotype groups, and in male and female mice. These findings, paired with the description of deglutition phenotypes at p21 and p35, provide a baseline understanding of tongue maturation phenotypes that occur in a mouse model of DS.

## Data Availability

The raw data supporting the conclusions of this article will be made available by the authors, without undue reservation. Microscopy data are publicly available on the SPARC Portal (RRID:SCR_017041) at the following DOI: https://doi.org/10.26275/ol8k-rau8.

## References

[1] Vorperian HK Kent RD Lee Y Buhr KA. Vowel production in children and adults with Down syndrome: fundamental and formant frequencies of the corner vowels. J Speech Lang Hear Res Apr. (2023) 66:1208–39. doi: 10.1044/2022_JSLHR-22-00510, PMID: 37015000 PMC10187968

[2] Wild A Vorperian HK Kent RD Bolt DM Austin D. Single-word speech intelligibility in children and adults with Down syndrome. Am J Speech Lang Pathol. (2018) 27:222–36. doi: 10.1044/2017_AJSLP-17-0002, PMID: 29214307 PMC5968330

[3] Kent RD Vorperian HK. Speech impairment in Down syndrome: a review. J Speech Lang Hear Res. (2013) 56:178–210. doi: 10.1044/1092-4388(2012/12-0148), PMID: 23275397 PMC3584188

[4] Donnelly LF Shott SR LaRose CR Chini BA Amin RS. Causes of persistent obstructive sleep apnea despite previous tonsillectomy and adenoidectomy in children with Down syndrome as depicted on static and dynamic cine MRI. AJR Am J Roentgenol. (2004) 183:175–81. doi: 10.2214/ajr.183.1.1830175, PMID: 15208134

[5] Mizuno K Ueda A. Development of sucking behavior in infants with Down’s syndrome. Acta Paediatr. (2001) 90:1384–8. doi: 10.1111/j.1651-2227.2001.tb01600.x, PMID: 11853333

[6] Kumin L Bahr DC. Patterns of feeding, eating, and drinking in young children with Down syndrome with oral motor concerns. Down Syndr Q. (1999) 1:1–8.

[7] Jackson A Maybee J Moran MK Wolter-Warmerdam K Hickey F. Clinical characteristics of dysphagia in children with Down syndrome. Dysphagia. (2016) 31:663–71. doi: 10.1007/s00455-016-9725-7, PMID: 27405422

[8] Eslick GD Talley NJ. Dysphagia: epidemiology, risk factors and impact on quality of life--a population-based study. Aliment Pharmacol Ther. (2008) 27:971–9. doi: 10.1111/j.1365-2036.2008.03664.x 18315591

[9] Feeney R Desha L Ziviani J Nicholson JM. Health-related quality-of-life of children with speech and language difficulties: a review of the literature. Int J Speech Lang Pathol. (2012) 14:59–72. doi: 10.3109/17549507.2011.604791, PMID: 21936757

[10] Delaney AL Arvedson JC. Development of swallowing and feeding: prenatal through first year of life. Dev Disabil Res Rev. (2008) 14:105–17. doi: 10.1002/ddrr.16, PMID: 18646020

[11] Iskander A Sanders I. Morphological comparison between neonatal and adult human tongues. Ann Otol Rhinol Laryngol. (2003) 112:768–76. doi: 10.1177/000348940311200905, PMID: 14535560

[12] Sanders I Mu L. A three-dimensional atlas of human tongue muscles. Anat Rec (Hoboken). (2013) 296:1102–14. doi: 10.1002/ar.22711, PMID: 23650264 PMC3687025

[13] Kayalioglu M Shcherbatyy V Seifi A Liu ZJ. Roles of intrinsic and extrinsic tongue muscles in feeding: electromyographic study in pigs. Arch Oral Biol. (2007) 52:786–96. doi: 10.1016/j.archoralbio.2007.01.004, PMID: 17350586 PMC2241921

[14] Denny M McGowan RS. Implications of peripheral muscular and anatomical development for the acquisition of lingual control for speech production: a review. Folia Phoniatr Logop. (2012) 64:105–15. doi: 10.1159/000338611, PMID: 22585234

[15] Yarom R Sagher U Havivi Y Peled IJ Wexler MR. Myofibers in tongues of Down’s syndrome. J Neurol Sci. (1986) 73:279–87. doi: 10.1016/0022-510X(86)90152-8, PMID: 2941522

[16] Cowley PM Keslacy S Middleton FA DeRuisseau LR Fernhall B Kanaley JA. Functional and biochemical characterization of soleus muscle in Down syndrome mice: insight into the muscle dysfunction seen in the human condition. Am J Physiol Regul Integr Comp Physiol. (2012) 303:R1251–60. doi: 10.1152/ajpregu.00312.2012 23115123

[17] Terry EE Zhang X Hoffmann C Hughes LD Lewis SA Li J. Transcriptional profiling reveals extraordinary diversity among skeletal muscle tissues. eLife. (2018) 7:613. doi: 10.7554/eLife.34613, PMID: 29809149 PMC6008051

[18] Talmadge RJ Roy RR Edgerton VR. Muscle fiber types and function. Curr Opin Rheumatol. (1993) 5:695–705. doi: 10.1097/00002281-199305060-00002 8117530

[19] Schiaffino S. Muscle fiber type diversity revealed by anti-myosin heavy chain antibodies. FEBS J. (2018) 285:3688–94. doi: 10.1111/febs.14502, PMID: 29761627

[20] Schiaffino S Reggiani C. Fiber types in mammalian skeletal muscles. Physiol Rev. (2011) 91:1447–531. doi: 10.1152/physrev.00031.2010 22013216

[21] Glass TJ Connor NP. Digastric muscle phenotypes of the Ts65Dn mouse model of Down syndrome. PLoS One. (2016) 11:e0158008. doi: 10.1371/journal.pone.0158008, PMID: 27336944 PMC4919106

[22] Okubo K Abe S Usami A Agematsu H Nakamura H Hashimoto M. Changes in muscle-fiber properties of the murine digastric muscle before and after weaning. Zool Sci. (2006) 23:1079–84. doi: 10.2108/zsj.23.1079, PMID: 17261921

[23] Suzuki K Abe S Kim HJ Usami A Iwanuma O Okubo H. Changes in the muscle fibre properties of the mouse temporal muscle after weaning. Anat Histol Embryol. (2007) 36:103–6. doi: 10.1111/j.1439-0264.2006.00729.x, PMID: 17371381

[24] Cullins MJ Connor NP. Alterations of intrinsic tongue muscle properties with aging. Muscle Nerve. (2017) 56:E119–25. doi: 10.1002/mus.25605, PMID: 28181263 PMC5550369

[25] Sanders I Mu L Amirali A Su H Sobotka S. The human tongue slows Down to speak: muscle fibers of the human tongue. Anat Rec (Hoboken). (2013) 296:1615–27. doi: 10.1002/ar.22755, PMID: 23929762 PMC3787083

[26] Maejima M Abe S Sakiyama K Agematsu H Hashimoto M Tamatsu Y. Changes in the properties of mouse tongue muscle fibres before and after weaning. Arch Oral Biol. (2005) 50:988–93. doi: 10.1016/j.archoralbio.2005.03.007, PMID: 15878764

[27] Yanagisawa N Abe S Agematsu H Sakiyama K Usami A Tamatsu Y. Myosin heavy chain composition of tongue muscle in microphthalmic (mi/mi) mice before and after weaning. Ann Anat. (2006) 188:329–36. doi: 10.1016/j.aanat.2006.02.004, PMID: 16856597

[28] Agbulut O Noirez P Beaumont F Butler-Browne G. Myosin heavy chain isoforms in postnatal muscle development of mice. Biol Cell. (2003) 95:399–406. doi: 10.1016/S0248-4900(03)00087-X, PMID: 14519557

[29] Lunteren EV Dick TE. Neural control of the respiratory muscles. Boca Raton, FL: CRC Press (1997). 336 p.

[30] Comparative Anatomy and Histology. A mouse and human atlas. Cambridge, MA: Academic Press (2012).

[31] Smith JC Goldberg SJ Shall MS. Phenotype and contractile properties of mammalian tongue muscles innervated by the hypoglossal nerve. Respir Physiol Neurobiol. (2005) 147:253–62. doi: 10.1016/j.resp.2005.02.016, PMID: 16087149

[32] Lever TE Braun SM Brooks RT Harris RA Littrell LL Neff RM. Adapting human videofluoroscopic swallow study methods to detect and characterize dysphagia in murine disease models. J Vis Exp. (2015) 97:52319. doi: 10.3791/52319-v PMC4401177 25866882

[33] Lever TE Gorsek A Cox KT O’Brien KF Capra NF Hough MS. An animal model of oral dysphagia in amyotrophic lateral sclerosis. Dysphagia. (2009) 24:180–95. doi: 10.1007/s00455-008-9190-z, PMID: 19107538

[34] Bailoo JD Voelkl B Varholick J Novak J Murphy E Rosso M. Effects of weaning age and housing conditions on phenotypic differences in mice. Sci Rep. (2020) 10:11684. doi: 10.1038/s41598-020-68549-3, PMID: 32669633 PMC7363894

[35] Grounds MD Radley HG Lynch GS Nagaraju K De Luca A. Towards developing standard operating procedures for pre-clinical testing in the mdx mouse model of Duchenne muscular dystrophy. Neurobiol Dis. (2008) 31:1–19. doi: 10.1016/j.nbd.2008.03.008, PMID: 18499465 PMC2518169

[36] Fox WM. Reflex-ontogeny and behavioural development of the mouse. Anim Behav. (1965) 13:234–IN5. doi: 10.1016/0003-3472(65)90041-2, PMID: 5835840

[37] Davisson MT Schmidt C Reeves RH Irving NG Akeson EC Harris BS. Segmental trisomy as a mouse model for Down syndrome. Prog Clin Biol Res. (1993) 384:117–33. PMID: 8115398

[38] Costa AC Stasko MR Schmidt C Davisson MT. Behavioral validation of the Ts65Dn mouse model for Down syndrome of a genetic background free of the retinal degeneration mutation Pde6b(rd1). Behav Brain Res. (2010) 206:52–62. doi: 10.1016/j.bbr.2009.08.034, PMID: 19720087 PMC2783207

[39] Glass TJ Valmadrid LCV Connor NP. The adult Ts65Dn mouse model of Down syndrome shows altered swallow function. Front Neurosci. (2019) 13:906. doi: 10.3389/fnins.2019.00906, PMID: 31555077 PMC6727863

[40] Billingsley CN Allen JR Baumann DD Deitz SL Blazek JD Newbauer A. Non-trisomic homeobox gene expression during craniofacial development in the Ts65Dn mouse model of Down syndrome. Am J Med Genet A. (2013) 161A:1866–74. doi: 10.1002/ajmg.a.36006, PMID: 23843306 PMC3729611

[41] Hill CA Reeves RH Richtsmeier JT. Effects of aneuploidy on skull growth in a mouse model of Down syndrome. J Anat. (2007) 210:394–405. doi: 10.1111/j.1469-7580.2007.00705.x, PMID: 17428201 PMC2100298

[42] Glass TJ Twadell SL Valmadrid LC Connor NP. Early impacts of modified food consistency on oromotor outcomes in mouse models of Down syndrome. Physiol Behav. (2019) 199:273–81. doi: 10.1016/j.physbeh.2018.11.031, PMID: 30496741 PMC6358162

[43] Glass T Hang K. *Myosin heavy chain (MyHC) 2a & 2b stain of mouse intrinsic tongue*. (2024). doi: 10.17504/protocols.io.8epv5rzjdg1b/v1

[44] Schindelin J Arganda-Carreras I Frise E Kaynig V Longair M Pietzsch T. Fiji: an open-source platform for biological-image analysis. Nat Methods. (2012) 9:676–82. doi: 10.1038/nmeth.2019 22743772 PMC3855844

[45] Davydova L Tkach G Tymoshenko A Moskalenko A Sikora V Kyptenko L. Anatomical and morphological aspects of papillae, epithelium, muscles, and glands of rats’ tongue: light, scanning, and transmission electron microscopic study. Interv Med Appl Sci. (2017) 9:168–77. doi: 10.1556/1646.9.2017.21, PMID: 29201443 PMC5700697

[46] Cullins MJ Krekeler BN Connor NP. Differential impact of tongue exercise on intrinsic lingual muscles. Laryngoscope. (2018) 128:2245–51. doi: 10.1002/lary.27044, PMID: 29243257 PMC6003827

[47] Zaidi FN Meadows P Jacobowitz O Davidson TM. Tongue anatomy and physiology, the scientific basis for a novel targeted neurostimulation system designed for the treatment of obstructive sleep apnea. Neuromodulation. (2013) 16:376–86. doi: 10.1111/j.1525-1403.2012.00514.x, PMID: 22938390

[48] Smith LR Barton ER. SMASH - semi-automatic muscle analysis using segmentation of histology: a MATLAB application. Skelet Muscle. (2014) 4:21. doi: 10.1186/2044-5040-4-21 25937889 PMC4417508

[49] Poskanzer SA Hobensack VL Ciciora SL Santoro SL. Feeding difficulty and gastrostomy tube placement in infants with Down syndrome. Eur J Pediatr. (2020) 179:909–17. doi: 10.1007/s00431-020-03591-x, PMID: 31984440

[50] Bull MJ Trotter T Santoro SL Christensen C Grout RW THE COUNCIL ON GENETICS. Health supervision for children and adolescents with Down syndrome. Pediatrics. (2022) 149:7010. doi: 10.1542/peds.2022-057010 35490285

[51] Allison Bechard GM. Leaving home: a study of laboratory mouse pup independence. Appl Anim Behav Sci. (2010) 125:181–8. doi: 10.1016/j.applanim.2010.04.006

[52] Zemel BS Pipan M Stallings VA Hall W Schadt K Freedman DS. Growth charts for children with Down syndrome in the United States. Pediatrics. (2015) 136:e1204–11. doi: 10.1542/peds.2015-1652, PMID: 26504127 PMC5451269

[53] Stanier MW Mount LE. Growth rate, food intake and body composition before and after weaning in strains of mice selected for mature body-weight. Br J Nutr. (1972) 28:307–25. doi: 10.1079/bjn19720041, PMID: 4673586

[54] Stanier MW Mount LE. Birth weight and growth rate during suckling and after weaning in strains of mice selected for mature body-weight. Proc Nutr Soc. (1972) 31:16A–7A. PMID: 5065513

[55] Hopman E Csizmadia CG Bastiani WF Engels QM De Graaf EA Le Cessie S. Eating habits of young children with Down syndrome in the Netherlands: adequate nutrient intakes but delayed introduction of solid food. J Am Diet Assoc. (1998) 98:790–4. doi: 10.1016/S0002-8223(98)00178-3, PMID: 9664921

[56] Homer E Carbajal P. Swallowing and feeding Services in the Schools: from therapy to the dinner table. Perspect Swallowing Swallowing Disord. (2015) 24:155–61. doi: 10.1044/sasd24.4.155

[57] Maksimoski M Li C. Surgical Management of Pediatric Obstructive Sleep Apnea beyond Tonsillectomy & adenoidectomy: Tongue Base and larynx. Otolaryngol Clin N Am. (2024) 57:431–45. doi: 10.1016/j.otc.2024.02.005, PMID: 38523050

[58] Kays SA Hind JA Gangnon RE Robbins J. Effects of dining on tongue endurance and swallowing-related outcomes. J Speech Lang Hear Res. (2010) 53:898–907. doi: 10.1044/1092-4388(2009/09-0048), PMID: 20689047 PMC3077124

[59] Brates D Molfenter S. The influence of age, eating a meal, and systematic fatigue on swallowing and mealtime parameters. Dysphagia. (2021) 36:1096–109. doi: 10.1007/s00455-020-10242-8 33479862

[60] Richtsmeier JT Baxter LL Reeves RH. Parallels of craniofacial maldevelopment in Down syndrome and Ts65Dn mice. Dev Dyn. (2000) 217:137–45. doi: 10.1002/(SICI)1097-0177(200002)217:2<137::AID-DVDY1>3.0.CO;2-N, PMID: 10706138

[61] Kaczorowska N Kaczorowski K Laskowska J Mikulewicz M. Down syndrome as a cause of abnormalities in the craniofacial region: a systematic literature review. Adv Clin Exp Med. (2019) 28:1587–92. doi: 10.17219/acem/112785, PMID: 31778604

[62] Guimaraes CV Donnelly LF Shott SR Amin RS Kalra M. Relative rather than absolute macroglossia in patients with Down syndrome: implications for treatment of obstructive sleep apnea. Pediatr Radiol. (2008) 38:1062–7. doi: 10.1007/s00247-008-0941-7, PMID: 18685841

[63] Sarver DC Xu C Velez LM Aja S Jaffe AE Seldin MM. Dysregulated systemic metabolism in a Down syndrome mouse model. Mol Metab. (2023) 68:101666. doi: 10.1016/j.molmet.2022.101666, PMID: 36587842 PMC9841171

[64] NIH. *The INCLUDE project research plan. (INvestigation of co-occurring conditions across the lifespan to understand Down syndrome)*. Available at: https://www.nih.gov/include-project/include-project-research-plan#background (Accessed February 11, 2019).

