# Supplementary material for: Developmental deglutition and intrinsic tongue muscle maturation phenotypes in the Ts65Dn mouse model of Down syndrome
Source: Front Neurol. 2024 Dec 11;15:1461682. doi: 10.3389/fneur.2024.1461682 (PMC11668655; doi:10.3389/fneur.2024.1461682)
Supplement: Supplementary file 1 [file Table_1.docx]

| **Intrinsic Tongue Region** | **Myofiber Measure** | **Age** | **Characteristic** | **β** | **95% CI** | **P value** |
| --- | --- | --- | --- | --- | --- | --- |
| **Anterior**  **Transverse muscle** | **minimum feret** | P7 | Genotype (Ts65Dn vs euploid) | -0.11 | -0.71, 0.50 | .729 |
|  |  |  | **Weight** | **0.41** | **0.13, 0.70** | **.005**** |
|  |  |  | Sex (Male vs Female) | -0.14 | -.63, 0.35 | .567 |
|  |  | P21-P22 | Genotype (Ts65Dn vs euploid) | -0.19 | -0.95, 0.57 | .620 |
|  |  |  | **Weight** | **0.17** | **0.00, 0.35** | **.049*** |
|  |  |  | Sex (Male vs Female) | -0.28 | -0.91, 0.36 | .385 |
|  |  | P35-P36 | Genotype (Ts65Dn vs euploid) | 0.38 | -0.37, 1.1 | .312 |
|  |  |  | Weight | 0.10 | -0.02, 0.22 | .109 |
|  |  |  | Sex (Male vs Female) | -0.17 | -0.88, 0.54 | .635 |
|  | **CSA** | P7 | Genotype (Ts65Dn vs euploid) | -3.4 | -18, 11 | .626 |
|  |  |  | **Weight** | **9.7** | **3.1, 16** | **.005**** |
|  |  |  | Sex (Male vs Female) | -3.0 | -14, 8.5 | .606 |
|  |  | P21-P22 | Genotype (Ts65Dn vs euploid) | -5.9 | -28, 16 | .583 |
|  |  |  | **Weight** | **5.6** | **0.69, 11** | **.026*** |
|  |  |  | Sex (Male vs Female) | -10 | -28, 8.1 | .274 |
|  |  | P35-P36 | Genotype (Ts65Dn vs euploid) | 7.8 | -14, 30 | .471 |
|  |  |  | Weight | 3.4 | -0.16, 6.9 | .061 |
|  |  |  | Sex (Male vs Female) | -5.0 | -26, 16 | .626 |
|  | **MyHC 2b+ CSA** | P7 | Genotype (Ts65Dn vs euploid) | -38 | -114, 38 | .309 |
|  |  |  | Weight | 18 | -20, 56 | .333 |
|  |  |  | Sex (Male vs Female) | -8.5 | -66, 49 | .766 |
|  |  | P21-P22 | Genotype (Ts65Dn vs euploid) | 1.5 | -24, 27 | .910 |
|  |  |  | **Weight** | **11** | **4.8, 16** | **<.001***** |
|  |  |  | Sex (Male vs Female) | -15 | -36, 6.9 | .178 |
|  |  | P35-P36 | Genotype (Ts65Dn vs euploid) | 6.2 | -20, 33 | .640 |
|  |  |  | Weight | 4.1 | -0.11, 8.4 | .056 |
|  |  |  | Sex (Male vs Female) | -12 | -37, 13 | .342 |
|  | **MyHC 2a+ CSA** | **P7** | **Genotype (Ts65Dn vs euploid)** | **29** | **9.1, 49** | **.006**** |
|  |  |  | **Weight** | **15** | **5.0, 24** | **.004**** |
|  |  |  | Sex (Male vs Female) | 8.6 | -8.4, 26 | .311 |
|  |  | P21-P22 | Genotype (Ts65Dn vs euploid) | 29 | -24, 81 | .271 |
|  |  |  | Weight | 8.8 | -2.5, 20 | .123 |
|  |  |  | Sex (Male vs Female) | 6.0 | -38, 50 | .784 |
|  |  | P35-P36 | Genotype (Ts65Dn vs euploid) | 19 | -86, 123 | .705 |
|  |  |  | Weight | 7.1 | -16, 31 | .524 |
|  |  |  | Sex (Male vs Female) | 25 | -85, 135 | .628 |

**Supplemental Table 1.** Subgroup analysis of Anterior Transverse Myofibers with co-variates of genotype, weight, and sex. * P < .05, ** P ≤ .01, *** P ≤ .001.

**Supplemental Table 2.** Subgroup analysis of Middle Transverse Myofibers with co-variates of genotype, weight, and sex. * P < .05, ** P ≤ .01, *** P ≤ .001.

| **Intrinsic Tongue Region** | **Myofiber Measure** | **Age** | **Characteristic** | **Β** | **95% CI** | **P value** |
| --- | --- | --- | --- | --- | --- | --- |
| **Middle**  **Transverse muscle** | **minimum feret** | P7 | Genotype (Ts65Dn vs euploid) | -0.77 | -1.7, 0.13 | .091 |
|  |  |  | Weight | 0.14 | -0.28, 0.56 | .498 |
|  |  |  | Sex (Male vs Female) | -0.22 | -0.95, 0.50 | .538 |
|  |  | P21-P22 | Genotype (Ts65Dn vs euploid) | -0.21 | -1.1, 0.66 | .625 |
|  |  |  | Weight | 0.19 | -0.01, 0.39 | .064 |
|  |  |  | Sex (Male vs Female) | -0.32 | -1.1, 0.43 | .400 |
|  |  | P35-P36 | Genotype (Ts65Dn vs euploid) | -0.20 | -1.1, 0.70 | .657 |
|  |  |  | Weight | 0.14 | -0.01, 0.28 | .060 |
|  |  |  | Sex (Male vs Female) | 0.02 | -0.81, 0.86 | .957 |
|  | **CSA** | P7 | Genotype (Ts65Dn vs euploid) | -25 | -50, 0.50 | .055 |
|  |  |  | Weight | 3.9 | -7.8, 16 | .506 |
|  |  |  | Sex (Male vs Female) | -7.1 | -27, 13 | .484 |
|  |  | P21-P22 | Genotype (Ts65Dn vs euploid) | -2.1 | -27, 23 | .863 |
|  |  |  | **Weight** | **6.4** | **0.65, 12** | **.030 *** |
|  |  |  | Sex (Male vs Female) | -12 | -33, 9.4 | .267 |
|  |  | P35-P36 | Genotype (Ts65Dn vs euploid) | -4.0 | -32, 24 | .777 |
|  |  |  | **Weight** | **4.8** | **0.23, 9.3** | **.040*** |
|  |  |  | Sex (Male vs Female) | .74 | -26, 27 | .956 |
|  | **MyHC 2b+ CSA** | P7 | Genotype (Ts65Dn vs euploid) | -55 | -142, 31 | 0.198 |
|  |  |  | Weight | 13 | -34, 60 | .566 |
|  |  |  | Sex (Male vs Female) | -43 | -115, 30 | .236 |
|  |  | P21-P22 | Genotype (Ts65Dn vs euploid) | -10 | -47, 26 | .577 |
|  |  |  | Weight | 8.0 | -0.34, 16 | .060 |
|  |  |  | Sex (Male vs Female) | -11 | -43, 20 | .467 |
|  |  | P35-P36 | Genotype (Ts65Dn vs euploid) | 4.0 | -34, 42 | .833 |
|  |  |  | Weight | 6.6 | 0.48, 13 | .035 |
|  |  |  | Sex (Male vs Female) | -2.6 | -38, 33 | .884 |
|  | **MyHC 2a+ CSA** | P7 | Genotype (Ts65Dn vs euploid) | -3.4 | -23, 16 | .726 |
|  |  |  | Weight | -1.6 | -11, 7.4 | .721 |
|  |  |  | Sex (Male vs Female) | -5.9 | -21, 9.6 | .446 |
|  |  | P21-P22 | Genotype (Ts65Dn vs euploid) | 19 | -1.4, 40 | .067 |
|  |  |  | **Weight** | **6.4** | **1.6, 11** | **.010**** |
|  |  |  | Sex (Male vs Female) | -5.2 | -23, 13 | .559 |
|  |  | P35-P36 | Genotype (Ts65Dn vs euploid) | 34 | -4.1, 72 | .079 |
|  |  |  | Weight | 1.4 | -4.8, 7.6 | .657 |
|  |  |  | Sex (Male vs Female) | 34 | -2.8, 70 | .069 |

| **Intrinsic Tongue Region** | **Myofiber Measure** | **Age** | **Characteristic** | **β** | **95% CI** | **P value** |
| --- | --- | --- | --- | --- | --- | --- |
| **Posterior**  **Transverse muscle** | **minimum feret** | P7 | Genotype (Ts65Dn vs euploid) | 0.53 | -0.66, 1.7 | .373 |
|  |  |  | **Weight** | **0.60** | **0.01, 1.2** | **.045*** |
|  |  |  | Sex (Male vs Female) | 0.25 | -0.72, 1.2 | .604 |
|  |  | P21-P22 | Genotype (Ts65Dn vs euploid) | -0.07 | -0.90, 0.77 | .868 |
|  |  |  | Weight | -0.06 | -0.25, 0.13 | .528 |
|  |  |  | Sex (Male vs Female) | -0.06 | -0.78, 0.65 | .861 |
|  |  | P35-P36 | Genotype (Ts65Dn vs euploid) | -0.21 | -1.3, 0.87 | .701 |
|  |  |  | Weight | 0.05 | -0.12, 0.23 | .530 |
|  |  |  | Sex (Male vs Female) | 0.36 | -0.65, 1.4 | .475 |
|  | **CSA** | P7 | Genotype (Ts65Dn vs euploid) | 5.9 | -32, 44 | .754 |
|  |  |  | Weight | 16 | -3.1, 34 | .099 |
|  |  |  | Sex (Male vs Female) | 2.4 | -29, 34 | .876 |
|  |  | P21-P22 | Genotype (Ts65Dn vs euploid) | -1.6 | -28, 25 | .904 |
|  |  |  | Weight | -1.5 | -7.5, 4.6 | .630 |
|  |  |  | Sex (Male vs Female) | -3.1 | -26, 20 | .785 |
|  |  | P35-P36 | Genotype (Ts65Dn vs euploid) | -0.86 | -40, 38 | .965 |
|  |  |  | Weight | 2.9 | -3.4, 9.1 | .357 |
|  |  |  | Sex (Male vs Female) | 7.0 | -29, 43 | .698 |
|  | **MyHC 2b+ CSA** | P7 | Genotype (Ts65Dn vs euploid) | 11 | -74, 96 | .781 |
|  |  |  | Weight | 28 | -7.2, 63 | .108 |
|  |  |  | Sex (Male vs Female) | 125 | 51, 199 | .003^#^ |
|  |  | P21-P22 | Genotype (Ts65Dn vs euploid) | 13 | -43, 69 | .647 |
|  |  |  | Weight | -4.8 | -18, 8.1 | .460 |
|  |  |  | Sex (Male vs Female) | -29 | -77, 20 | .239 |
|  |  | P35-P36 | Genotype (Ts65Dn vs euploid) | -3.0 | -68, 62 | .926 |
|  |  |  | Weight | 6.9 | -3.6, 17 | .192 |
|  |  |  | Sex (Male vs Female) | -11 | -72, 50 | .712 |
|  | **MyHC 2a+ CSA** | P7 | Genotype (Ts65Dn vs euploid) | 2.1 | -25, 29 | .875 |
|  |  |  | Weight | 12 | -1.3, 26 | .074 |
|  |  |  | Sex (Male vs Female) | -6.7 | -29, 16 | .551 |
|  |  | P21-P22 | Genotype (Ts65Dn vs euploid) | -11 | -35, 13 | .352 |
|  |  |  | Weight | -1.5 | -6.9, 3.9 | .582 |
|  |  |  | Sex (Male vs Female) | -11 | -32, 8.9 | .263 |
|  |  | **P35-P36** | **Genotype (Ts65Dn vs euploid)** | **57** | **13, 101** | **.012*** |
|  |  |  | Weight | 3.2 | -3.9, 10 | .370 |
|  |  |  | Sex (Male vs Female) | -14 | -55, 27 | .499 |

**Supplemental Table 3.** Subgroup analysis of Posterior Transverse Myofibers with co-variates of genotype, weight, and sex. * P < .05, ‘#’ indicates finding may be spurious due to exceptionally low numbers of positive myofibers in the experimental subgroup.
