# Supplementary material for: Developmental deglutition and intrinsic tongue muscle maturation phenotypes in the Ts65Dn mouse model of Down syndrome
Source: Front Neurol. 2024 Dec 11;15:1461682. doi: 10.3389/fneur.2024.1461682 (PMC11668655; doi:10.3389/fneur.2024.1461682)
Supplement: Supplementary file 2 [file Data_Sheet_1.docx]

**
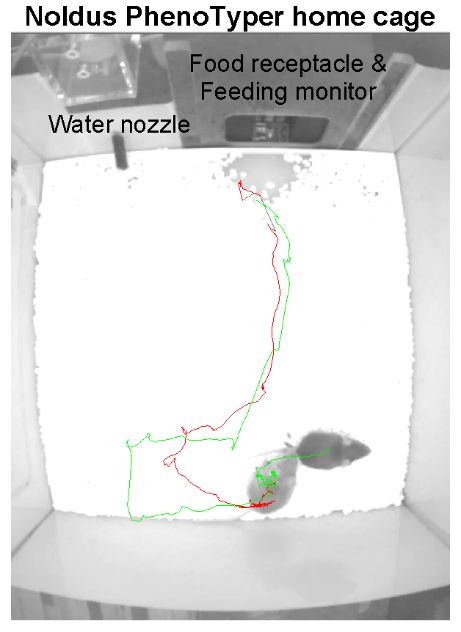
Figure S1.** An overhead view of the Noldus PhenoTyper home cage (floor area of 12x12 inches) used to evaluate eating and drinking behaviors, with automated mouse tracking through the Ethovision XT software. Modifications from the manufacturer default included insulation added to the shaft of the water nozzle to minimize artifactual signal from occasional contact between the mouse and the sides of the nozzle, as well as the creation of a raised floor to bring the mice close enough to the food and water to ensure rearing was not required.

**
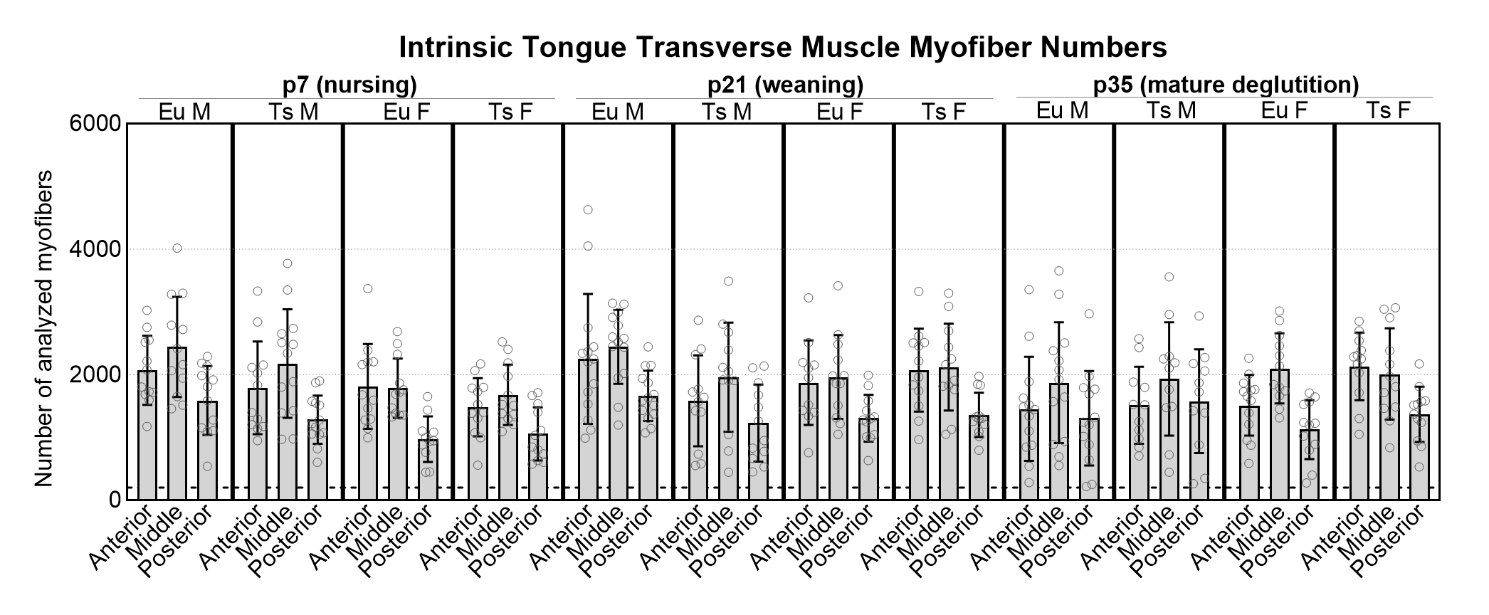
**

**Figure S2.** Data points indicate the number of myofibers analyzed in each anatomical region of the transverse muscle. Each data point indicates the number of myofibers analyzed in one mouse. Bars indicate mean and SD. The dark dotted line on the y-axis indicates 200 myofibers, which was set as the minimum number needed for inclusion in the study. The numbers of fibers analyzed may have been smaller in some cases than the numbers of fibers present, because fibers that were compromised or undetected due to mechanical damage or imaging artifacts were omitted from analysis.


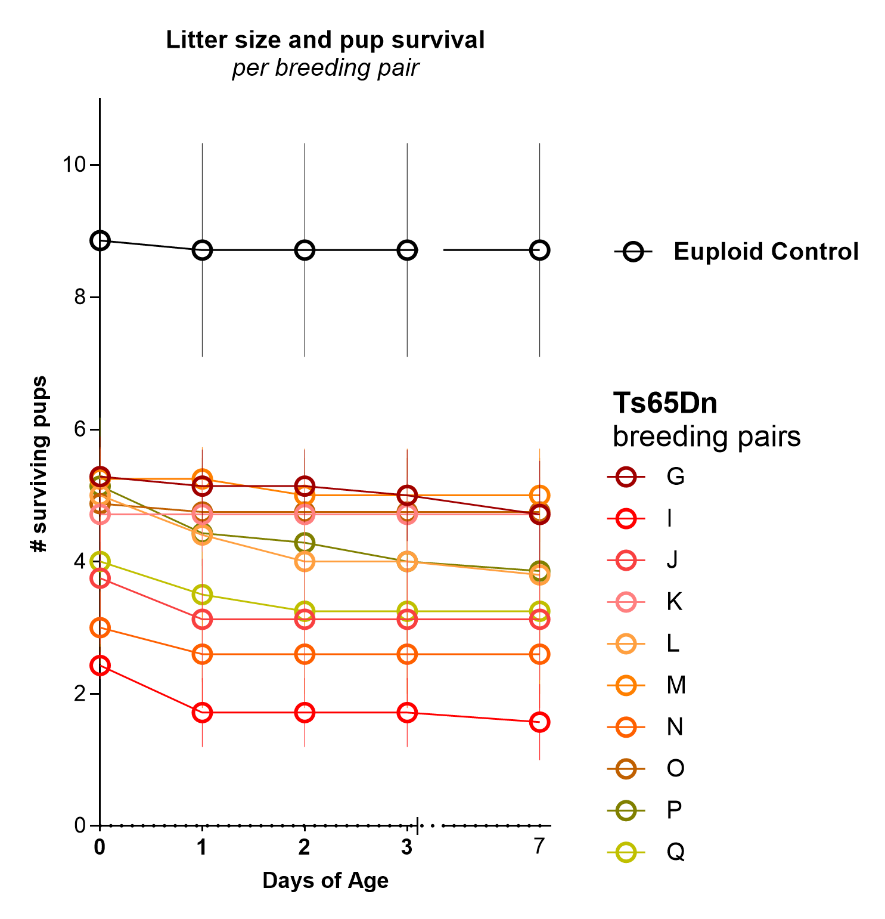


**Figure S3.** Pup survival was tracked daily from p0 to p7 for a euploid control breeding pair (producing litters of all euploid progeny), and Ts65Dn breeder pairs that were arbitrarily assigned alphabetic identifiers (producing litters comprised of both Ts65Dn and euploid progeny). The majority of spontaneous mortality occurred prior to p1. Graph indicates group means and SEM. N=4-8 litters per breeding pair. While the incidence of mortality at ages above p7 was not specifically tracked in data collected for this study, a low level of spontaneous mortality was incidentally observed to also occasionally occur between p7 and p21.
